# Discovery of novel dual adenosine A_1_/A_2A_ receptor antagonists using deep learning, pharmacophore modeling and molecular docking

**DOI:** 10.1371/journal.pcbi.1008821

**Published:** 2021-03-19

**Authors:** Mukuo Wang, Shujing Hou, Yu Wei, Dongmei Li, Jianping Lin

**Affiliations:** 1 State Key Laboratory of Medicinal Chemical Biology, College of Pharmacy and Tianjin Key Laboratory of Molecular Drug Research, Nankai University, Tianjin, China; 2 Biodesign Center, Tianjin Institute of Industrial Biotechnology, Chinese Academy of Sciences, Tianjin, China; 3 Platform of Pharmaceutical Intelligence, Tianjin International Joint Academy of Biomedicine, Tianjin, China; Icahn School of Medicine at Mount Sinai, UNITED STATES

## Abstract

Adenosine receptors (ARs) have been demonstrated to be potential therapeutic targets against Parkinson’s disease (PD). In the present study, we describe a multistage virtual screening approach that identifies dual adenosine A_1_ and A_2A_ receptor antagonists using deep learning, pharmacophore models, and molecular docking methods. Nineteen hits from the ChemDiv library containing 1,178,506 compounds were selected and further tested by *in vitro* assays (cAMP functional assay and radioligand binding assay); of these hits, two compounds (C8 and C9) with 1,2,4-triazole scaffolds possessing the most potent binding affinity and antagonistic activity for A_1_/A_2A_ ARs at the nanomolar level (pK_i_ of 7.16–7.49 and pIC_50_ of 6.31–6.78) were identified. Further molecular dynamics (MD) simulations suggested similarly strong binding interactions of the complexes between the A_1_/A_2A_ ARs and two compounds (C8 and C9). Notably, the 1,2,4-triazole derivatives (compounds C8 and C9) were identified as the most potent dual A_1_/A_2A_ AR antagonists in our study and could serve as a basis for further development. The effective multistage screening approach developed in this study can be utilized to identify potent ligands for other drug targets.

## Author summary

Parkinson’s disease (PD) is a long-term degenerative disorder of the central nervous system. Dual adenosine A_1_/A_2A_ receptor antagonists provide a possible effective therapy that can simultaneously solve motor symptoms and nonmotor symptoms in the medical treatment of PD. Here, we applied a multistage virtual screening approach involving deep learning, pharmacophore models, and molecular docking methods to the ChemDiv library, which contains 1,178,506 compounds, and identified two 1,2,4-triazole derivatives as dual adenosine A_1_ and A_2A_ receptor antagonists. We used a cAMP functional assay and radioligand binding assay to evaluate the biological activity and binding affinities towards adenosine A_1_ and A_2A_ receptor of 8 selected compounds, respectively. The molecular dynamics (MD) simulations showed similarly strong binding interactions of the complexes between the adenosine A_1_/A_2A_ receptors and the compounds. The results showed that the multistage virtual screening approach can effectively identify potent dual adenosine A_1_/A_2A_ receptor antagonists. The multistage screening approach offers an alternative and effective strategy for the identification of potent ligands for other drug targets.

This is a *PLOS Computational Biology* Methods paper.

## Introduction

Parkinson’s disease (PD) is a common and complex neurodegenerative disorder that is characterized by the early prominent death of dopaminergic neurons in the substantia nigra pars compacta and the abnormal aggregation of the α-synuclein protein, called Lewy bodies and Lewy neurites [1]. Decreased dopamine in the basal ganglia can cause classical motor symptoms [2], including bradykinesia, resting tremors, and postural instability [3], and nonmotor symptoms, including constipation, depression, sleep disturbance, apathy, hallucinations and dementia [4]. L-3,4-Dihydroxyphenylalanine (L-dopa), which is the direct precursor of dopamine, is commonly used as dopamine replacement therapy in the treatment of PD motor symptoms [5]. Although L-dopa can help relieve PD motor symptoms, its chronic use may cause side effects such as motor complications (motor fluctuations and dyskinesias) [6]. In addition, dopamine receptor agonists, catechol O-methyltransferase inhibitors, monoamine oxidase B (MAOB) inhibitors, amantadine and anticholinergic drugs are available on the market for the treatment of PD [7,8]. However, these drugs are mainly used to replace the concentration and/or effect of dopamine in the brain and only solve the motor symptoms but not the nonmotor symptoms [9]. Therefore, it is necessary to develop effective therapies that simultaneously solve the motor symptoms and nonmotor symptoms in medical treatments of PD.

In recent years, adenosine receptor (AR) antagonists have attracted much attention in the development of nondopaminergic therapies for the treatment of PD. Interestingly, high-resolution structures of A_1_ and A_2A_ adenosine receptors are available [10–20], providing an excellent opportunity for structure-based drug design (SBDD). Thus, virtual screening efforts have identified potent antagonists [21–23]. Among the four human adenosine receptors (A_1_, A_2A_, A_2B_ and A_3_) [24], the A_2A_ receptor, which is a potential target for the treatment of PD, has been intensively studied. A_2A_AR antagonists have been developed as a potential class of nondopaminergic antiparkinsonian agents that can relieve patients of symptoms of depression (nonmotor symptom of PD) [25]. The A_2A_AR antagonist KW-6002 has been proven to have antidepressant activity in the forced swim test and tail suspension test in rodents [26,27]. In addition, epidemiological and experimental data indicate that adenosine A_2A_ receptor antagonists have neuroprotective effects [28]. Compared with A_2A_AR, A_1_AR is more widely distributed in the central nervous system. A_1_AR is highly expressed in brain regions, including the hippocampus and prefrontal cortex. These regions are important for emotion and cognitive function. Furthermore, since A_1_AR antagonists can increase the release of acetylcholine and glutamate, and improve cognitive dysfunction [29], A_1_AR antagonism may improve the cognitive deficits experienced in PD as illustrated in animals studies [30].

**Fig 1:**
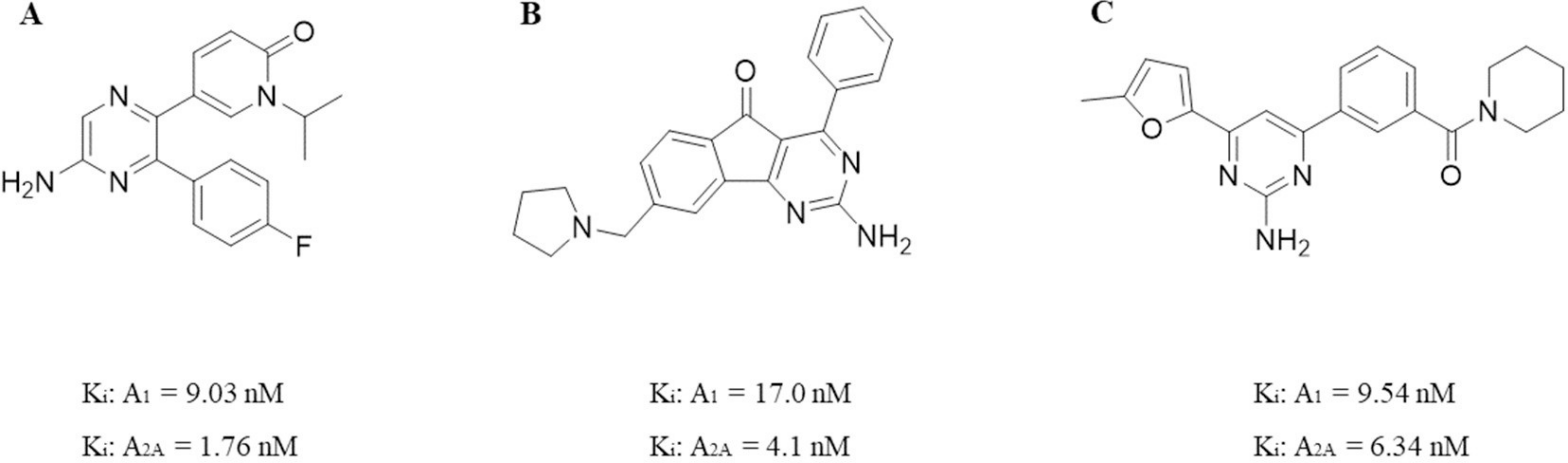
Structures of ASP5854 (A), 2-amino-4-phenyl-8-(pyrrolidin-1-ylmethyl)-5H-indeno[1,2-d]pyrimidin-5-one (B) and 4-(5-methylfuran-2-yl)-6-[3-(piperidine-1-carbonyl)phenyl]pyrimidin-2-amine (C), which were found to be dual A_1_/A_2A_ AR antagonists.

Among the adenosine receptor antagonists, caffeine, which is a xanthine derivative, has been found to be a nonselective A_1_AR and A_2A_AR antagonist. Epidemiological research has shown that a correlation exists between the intake of coffee or caffeine and a reduced risk of PD [31]. Subsequently, several dual A_1_/A_2A_ AR antagonists were further developed and proven to not only improve dyskinesia but also enhance cognition and play a neuroprotective role through the antagonistic effect of A_2A_AR [3,32,33]. Dual A_1_/A_2A_ AR antagonists may display synergistic motor activation. A_1_AR antagonism promotes presynaptic dopamine release, while A_2A_AR antagonism promotes postsynaptic dopamine release [34]. In conclusion, dual A_1_/A_2A_ AR antagonists not only treat the motor symptoms of PD and have neuroprotective effects but may also improve nonmotor symptoms [35]. In 2007, Mihara et al. discovered the dual A_1_/A_2A_ AR antagonist 5-[5-amino-3-(4-fluorophenyl)pyrazin-2-yl]-1-isopropylpyridine-2(1H)-one (ASP5854) (Fig 1A), which showed high affinity for human A_1_ and A_2A_ receptors with K_i_ values of 9.03 nM and 1.76 nM, respectively [36]. In 2008, ASP5854 was further confirmed to have activity comparable to that of existing anti-Parkinson’s disease drugs, but no further research investigating its safety was conducted [37]. In 2010, Shook et al. designed and synthesized an arylindenopyrimidine (2-amino-4-phenyl-8-(pyrrolidin-1-ylmethyl)-5H-indeno[1,2-d]pyrimidin-5-one, Fig 1B) derivative as a dual A_1_/A_2A_ AR antagonist that has excellent activity in various animal models of PD after oral administration, but further studies revealed that it was genotoxic [3]. In 2015, Robinson et al. characterized a small set of 2-aminopyridines as dual A_1_/A_2A_ AR antagonists. These compounds are considered useful for motor diseases, such as PD. The most potent compound found from this campaign was 4-(5-methylfuran-2-yl)-6-[3-(piperidine-1-carbonyl)phenyl]pyrimidin-2-amine (Fig 1C), which was able to bind A_1_ and A_2A_ receptors with K_i_ values of 9.54 nM and 6.34 nM, respectively [38,39]. Therefore, the identification of novel dual A_1_/A_2A_ AR antagonists is of great significance for the development of novel agents for the treatment of PD [40].

Deep learning (DL), or deep neural networks, has been developed based on artificial neural networks (ANNs), which are inspired by the biological structure and function of the brain. To date, several DL frameworks, such as deep neural networks (DNNs), convolutional neural networks (CNNs), deep belief networks (DBNs), and recurrent neural networks (RNNs), have been developed. Currently, the deep learning procedure, which is the dominant component of machine learning (ML) in artificial intelligence, has emerged as a vital tool for expediting the *in silico* screening of potential hits [41]. Unterthiner et al. showed that the efficacy of DL in virtual drug screening was better than that of seven other screening methods using ChEMBL benchmark data [42]. Lenselink et al. demonstrated that the screening performance of DNNs outperformed that of other machine learning methods such as support vector machines, random forests, naïve Bayes and logistic regression models [43]. Bilsland et al. trained an ANN classification model to screen for senescence-inducing compounds from a library of ~2 M lead-like compounds and identified a benzimidazolone compound with a low micromolar IC_50_ using *in vitro* assays as a potential hit for the development of selective cell cycle inhibitors [44]. Wallach et al. used CNN to design a structure-based model for the prediction of the bioactivity of small molecules for drug development and successfully predicted new active molecules for targets without known modulators [45]. Recently, Rifaioglu et al. developed DEEPScreen, which is a novel DTI prediction system based on deep convolutional neural networks that can predict new interactions between the drug cladribine and JAK proteins as confirmed by *in vitro* experiments using cancer cells [46]. Overall, deep learning plays an increasingly important role in modern drug discovery.

To date, the discovery of dual A_1_/A_2A_ AR antagonists has been achieved through structural modification of specific scaffolds to obtain new compounds, while a multistage virtual screening approach combining ligand-based and structural-based methods has rarely been used. Although the rapid development of the pharmacophore [47] and molecular docking [48] methods enabled the screening of very large databases containing billions of diverse molecules, these methods are individually unable to achieve perfect effects. For example, the inaccurate scoring functions implemented in docking methods often lead to a low hit rate and a high false positive rate. In this study, a multistage virtual screening system consisting of deep learning, pharmacophore models, and molecular docking methods was used to identify novel dual A_1_/A_2A_ AR antagonists from the ChemDiv database. The ChemDiv collection consists of more than 14,000 chemical families (chemotypes) and 1,250,000 diverse drug-like small molecules. Nineteen compounds were selected and tested to determine their A_1_AR and A_2A_AR functional activities; of these compounds, five compounds were found to have dual A_1_/A_2A_ AR antagonistic activity through cAMP functional assays and radioligand binding assays. Among the five compounds found to have dual A_1_/A_2A_ AR antagonistic activity, compounds C8 and C9, which contained a 1,2,4-triazole scaffold, showed the highest antagonistic activity, reaching nanomolar inhibition.

## Results and discussion

### Construction and validation of the DNN and CNN models

In this study, we used the self-developed Python script based on the algorithm from reference 50 to construct the DNN and CNN classification models of the dual A_1_/A_2A_ AR antagonists using a library containing 310 bioactive dual A_1_/A_2A_ antagonists (K_i_ < 40 nM) and 405 nonbioactive antagonists (K_i_ > 1000 nM). The models used the extended connectivity fingerprint 4 (ECFP4) [49] and neural fingerprint (NFP) [50]. Six evaluation indicators, including sensitivity (SE), specificity (SP), prediction accuracy of active molecules (Q+), prediction accuracy of inactive molecules (Q-), Matthews correlation coefficient (MCC) and area under the curve (AUC), were used to evaluate the classification ability of the models.

To assess the impact of the different batch sizes on the performance of the DNN and CNN classification models, we constructed the DNN and CNN models using batch sizes from 50 to 300 with an interval of 50. Table 1 lists the statistical evaluation results of the DNN classification models based on the ECFP4 of the test set under different batch sizes. All DNN classification models show very good SE, SP, Q+, Q-, MCC and AUC values. Among the six DNN classification models, Model_D5 based on ECFP4 has the best prediction ability, with an MCC of 0.891 and an AUC of 0.997. The optimized hyperparameters of Model_D5 which had the best performance, are as follows: batch size = 250, learning rate = 0.001, num_epochs (number of training epochs) = 500, dropout = 0.2, L2_regulation_type (L2 regularization parameters) = 0.0001, three hidden layers with layer widths of 3000, 2000 and 1000, and a final fully connected layer that uses the Softmax algorithm to produce classification results.

**Table 1:** Test results of the DNN classification model under different batch sizes.

DNN Model	Batch size	SE	SP	Q+	Q-	MCC	AUC
Model_D1	50	1	0.901	0.818	1	0.859	0.99
Model_D2	100	0.972	0.901	0.814	0.986	0.836	0.989
Model_D3	150	0.972	0.827	0.714	0.985	0.748	0.99
Model_D4	200	1	0.889	0.8	1	0.843	0.993
Model_D5	250	1	0.926	0.857	1	0.891	0.997
Model_D6	300	1	0.889	0.8	1	0.843	0.978

We further analyzed the performance of the CNN models in predicting dual A_1_/A_2A_ AR antagonists. The statistical evaluation of six CNN models based on NFP is shown in Table 2. The table shows that the batch size has a great impact on the prediction ability of the CNN classification models. Model_C6, which has a batch size of 300, shows the lowest MCC and AUC values, while Model _C4, which has a batch size of 200, shows the highest MCC and AUC values. The optimized hyperparameters of Model_C4, which has the best performance are as follows: learning rate = 0.001, num_epochs (number of training epochs) = 500, batch size = 200, L2_regulation_type (L2 regularization parameters) = 0.0001, and fingerprint_network_architecture (convolutional layers) = 5. Based on the evaluation results of the DNN and CNN classification models, Model_D5 and Model_C4, which had the best performance, were selected, and we conducted the following virtual screening of dual A_1_/A_2A_ AR antagonists.

**Table 2:** Test results of the CNN classification model under different batch sizes.

CNN Model	Batch size	SE	SP	Q+	Q-	MCC	AUC
Model_C1	50	0.861	0.877	0.756	0.934	0.714	0.941
Model_C2	100	0.972	0.914	0.833	0.987	0.852	0.986
Model_C3	150	0.944	0.926	0.85	0.974	0.847	0.977
Model_C4	200	1	0.926	0.857	1	0.891	0.986
Model_C5	250	0.972	0.926	0.854	0.987	0.869	0.985
Model_C6	300	1	0.185	0.353	1	0.256	0.791

### Pharmacophore model generation and validation

**Fig 2:**
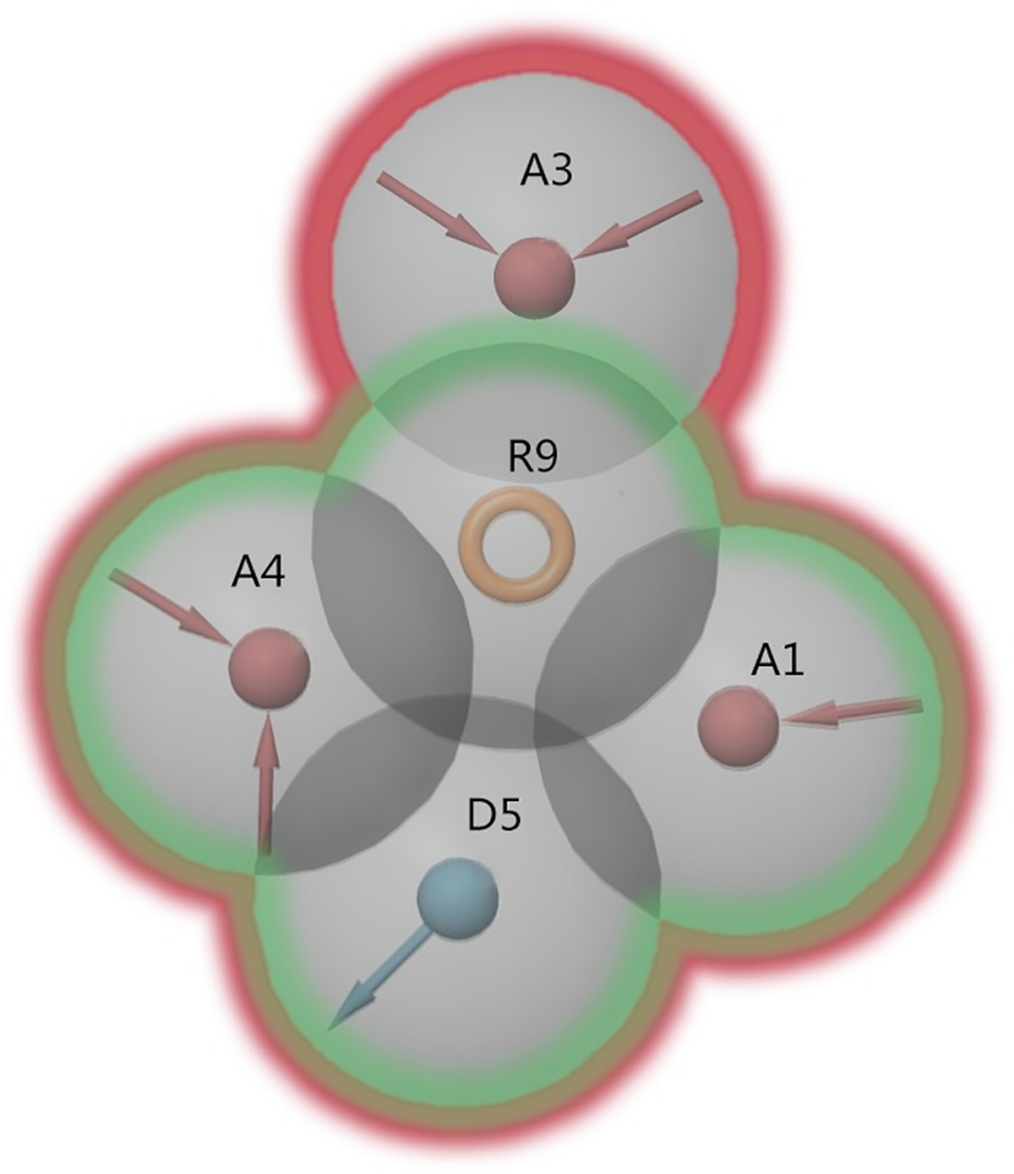
Pharmacophore features of pharmacophore hypotheses AADR_4 and AAADR_1. AADR_4 is displayed in green, and AAADR_1 is displayed in red.

A training set containing 14 active ingredients (S1 Table) was used to generate the dual A_1_/A_2A_ AR antagonist pharmacophore models. Eleven hypotheses were generated, which matched 9–13 of the 14 activities. To evaluate pharmacophore hypotheses, a validation set of 42 dual antagonists of the A_1_AR and A_2A_AR subtypes and 913 decoy compounds was used to explore the ability of the pharmacophore hypothesis to distinguish the dual A_1_/A_2A_ AR antagonists from the decoys. The statistical results of predicting the dual antagonists in the validation set are summarized in Table 3. The EF1% (enrichment factor in the top 1% of compounds screened) and BEDROC (α-160.9) (Boltzmann-enhanced discrimination of receiver operating characteristic) were used as “early recognition” metrics [51]. As shown in Fig 2, the AADR_4 and AAADR_1 hypotheses shared the same four pharmacophore sites including two hydrogen bond acceptors (A), one hydrogen donor (D) and one aromatic ring (R); however, the AAADR_1 hypothesis contains one additional hydrogen bond acceptor at the far end. When a minimum of four sites of the AAADR_1 model were used for screening (including the AADR_4 model), the most dual A_1_/A_2A_ AR antagonists (39 of 42) were retrieved from the validation set, corresponding to an ROC of 0.89. Finally, the five-pharmacophore-site (AAADR_1) hypotheses, which matched 12 of the 14 active ingredients in the training set, were selected for further virtual screening.

**Table 3:** Validation of the pharmacophore hypotheses.

Hypothesis	PhaseHypoScore	EF1%^a^	BEDROC (α-160.9)^b^	ROC^c^	AUAC^d^	Total Actives	Ranked Actives	Matches
AADR_4	0.69	22.74	1	0.83	0.90	42	35	4 of 4
AAADR_1	0.65	13.64	0.73	0.89	0.90	42	39	4 of 5
AADR_5	0.65	22.74	1	0.83	0.90	42	35	4 of 4
AADR_1	0.64	22.74	1	0.88	0.92	42	37	4 of 4
AAAR_1	0.63	13.64	0.68	0.77	0.83	42	34	4 of 4
AADR_2	0.61	22.74	1	0.81	0.89	42	34	4 of 4
ADHR_1	0.6	22.74	0.97	0.50	0.74	42	21	4 of 4
AARR_1	0.56	13.64	0.64	0.73	0.80	42	33	4 of 4
ADRR_2	0.5	22.74	0.98	0.57	0.77	42	24	4 of 4
ADRR_1	0.5	22.74	0.99	0.69	0.83	42	29	4 of 4
AADR_3	0.48	22.74	1	0.90	0.93	42	38	4 of 4

a EF1%: enrichment factor at 1% of the validation set.

b BEDROC (α-160.9): Boltzmann-enhanced discrimination of receiver operating characteristics.

c ROC: receiver operating characteristic curve value.

d AUAC: area under the accumulation curve.

### Virtual screening

**Fig 3:**
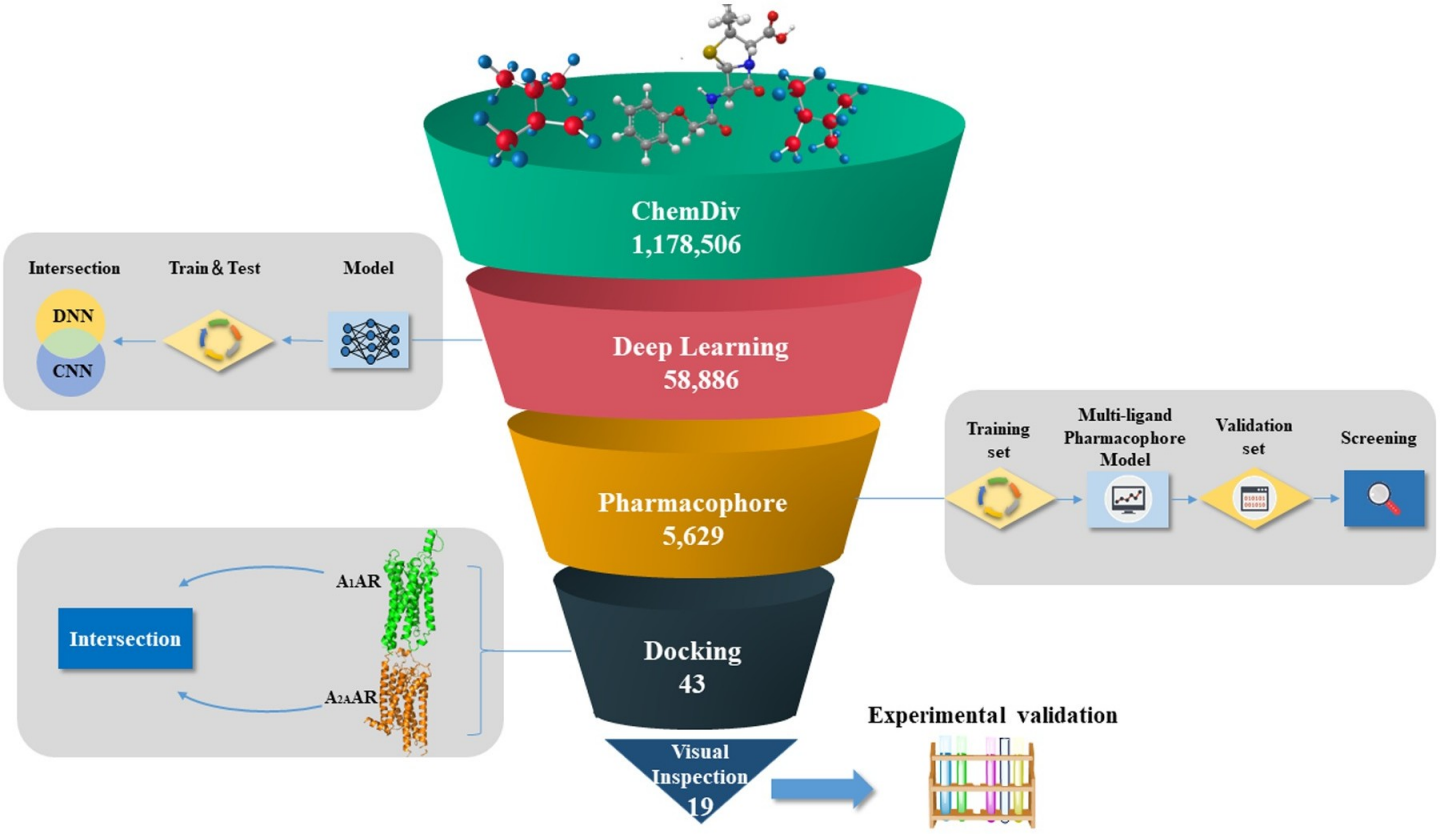
Schematic overview of the discovery of dual A_1_/A_2A_ AR antagonists by performing multistage virtual screening against the ChemDiv library.

Our previous study showed that a multistage approach sequentially integrating machine learning classification models and pharmacophore and molecular docking methods is efficient in identifying bioactive compounds from chemical databases [52]. In this study, the performance of the hierarchical multistage virtual screening approach was evaluated through a validation set containing 433 bioactive dual A_1_/A_2A_ AR antagonists (40 nM < K_i_ <600 nM) and 11605 decoys (S2 Table). Then, DNN and CNN classification models, a pharmacophore model (AAADR_1) and molecular docking were applied for the discovery of dual A_1_/A_2A_ AR antagonists by performing multistage virtual screening against the ChemDiv library (1,178,506 compounds) (Fig 3). In the first stage, the DNN and CNN classification models were separately utilized to filter 1,178,506 compounds. Initially, 209,585 and 171,233 compounds passed the filter that applied the DNN and CNN classification models, respectively. In total, 58,886 compounds simultaneously predicted by the DNN and CNN classification models were retained. In the second stage, a pharmacophore model was applied to remove the compounds that were not adequate for a minimum of four sites, and 5,629 of the 58,886 compounds that matched the requirements of the AAADR_1 model were retained. In the third stage, the 5,629 compounds were subjected to molecular docking screening based on the X-ray structures of A_1_AR (PDB ID 5N2S) and A_2A_AR (PDB ID 5IU4) using Glide HTVS, SP and XP functions. After the XP molecular docking, 43 compounds that simultaneously bind A_1_ and A_2A_ ARs remained. Based on the assessment of chemical-protein interactions by visual inspection, 19 of the 43 compounds that matched the four pharmacophore sites (S1 Fig) were finally selected for an in vitro evaluation of their bioactivity towards A_1_ and A_2A_ ARs. Fig 3 shows a schematic workflow of our multistage virtual screening methods.

### Biological evaluation

#### Functional experiments

**Fig 4:**
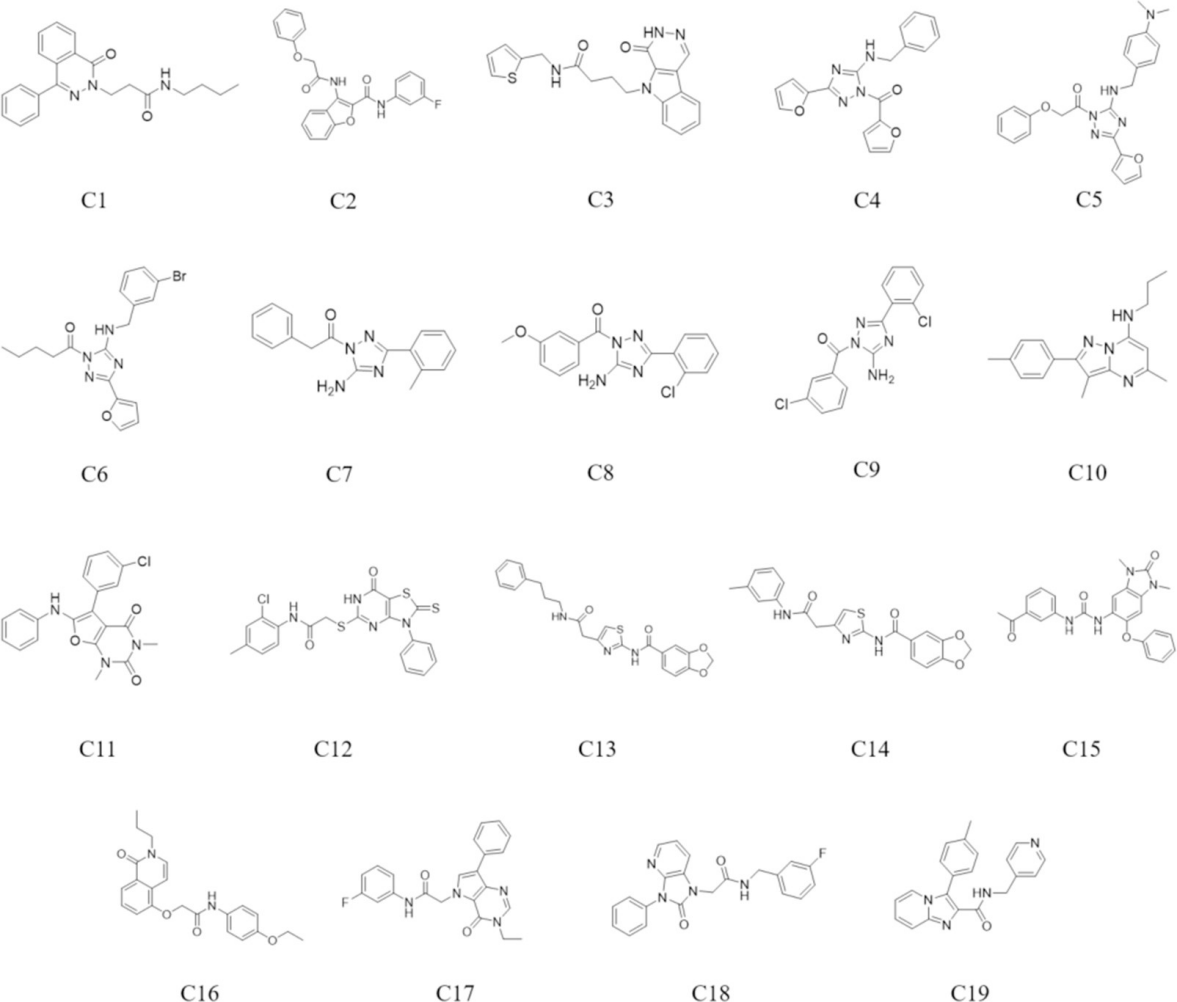
Chemical structures of compounds C1—C19.

Initially, the biological activity of selected compounds C1 –C19 towards A_1_ and A_2A_ ARs was evaluated by a cAMP functional assay. The potency of the antagonists at the A_1_ and A_2A_ ARs is depicted in Table 4, which shows that eight compounds (C4, C7, C8, C9, C10, C15, C17 and C19) of the 19 compounds (Fig 4) display potent antagonistic activity against A_1_AR with pIC_50_ values ranging from 4.28 to 6.78. Regarding the eight compounds, the pIC_50_ values at A_2A_AR were subsequently determined. Among all eight compounds, five compounds possessed antagonistic activity towards A_2A_AR with pIC_50_ values of 4.20–6.44. The concentration-response curves of the eight compounds in the cAMP assay are depicted in S2 and S3 Figs. Notably, compounds C8 and C9 have the most potent antagonistic activity towards A_1_/A_2A_ ARs with pIC_50_ values in the range of 6.31–6.78, while C15 has the weakest antagonistic activity towards A_1_/A_2A_ ARs with pIC_50_ values ranging between 5.10–5.35.

**Table 4:** Potencies and binding activities at human A_1_ and A_2A_ ARs of compounds C1 –C19. A_1_/A_2A_AR Affinity Experiments.

Compound number	Compound ID	cAMP assays (pIC_50_)		Binding affinities (pK_i_)		Pharmacophore matching
		A_1_	A_2A_	A_1_	A_2A_	
C1	6016–3052	< 4	--	--	--	4
C2	C686-0670	< 4	--	--	--	4
C3	C884-2451	< 4	--	--	--	4
C4	**D116-0057**	5.51	< 4	5.68	4.71	4
C5	D331-0235	NAN	--	--	--	4
C6	D331-0346	NAN	--	--	--	4
C7	**D481-1843**	5.72	< 4	5.98	5.24	4
C8	**D481-2135**	6.78	6.44	7.19	7.29	4
C9	**D481-2142**	6.72	6.31	7.49	7.16	4
C10	**D503-0436**	6.46	5.00	6.77	5.08	4
C11	F026-0231	< 4	--	--	--	4
C12	F092-0415	NAN	--	--	--	4
C13	F186-0821	< 4	--	--	--	4
C14	F186-0830	< 4	--	--	--	4
C15	**G433-0400**	5.35	5.10	5.54	< 4	4
C16	G856-9311	< 4	--	--	--	4
C17	**L935-0138**	6.20	4.20	6.28	< 4	4
C18	P072-0704	< 4	--	--	--	4
C19	**P433-0232**	4.28	< 4	4.97	4.93	4
	DPCPX	--	--	8.60	--	
	ZM241385	--	--	--	8.70	

#### A_1_/A_2A_AR affinity experiments

**Fig 5:**
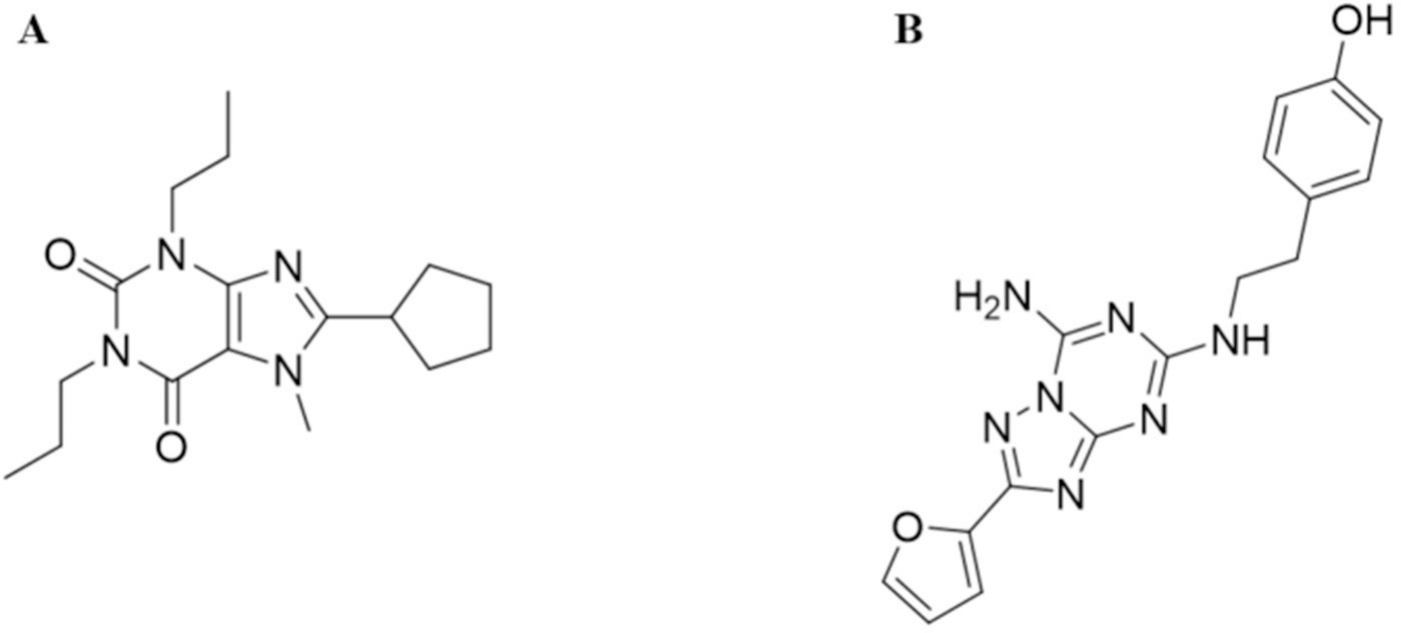
Chemical structures of DPCPX (A) and ZM241385 (B).

All eight compounds with potent antagonistic activity against A_1_/A_2A_ ARs were further evaluated to determine their binding affinity against A_1_ and A_2A_ ARs by radioligand binding assays using the labeled A_1_AR antagonist 8-cyclopentyl-1,3-dipropylxanthine (DPCPX) (Fig 5A) and A_2A_AR antagonist 4-[2-[[7-amino-2- (furyl)1,2,4-triazolo[2,3-a]1,3,5-triazin-5-yl]-amino]ethyl]phenol (ZM241385) (Fig 5B). The concentration-response curves of the eight compounds in the radioligand binding assay are depicted in S4 and S5 Figs. Table 4 shows that compounds C8 and C9 also possess the highest binding affinity for A_1_/A_2A_ ARs with K_i_ values at the nanomolar level (pK_i_ values in the range 7.16–7.49), which is consistent with the highest antagonistic potency with pIC_50_ values in the range 6.31–6.78. Overall, these experiments confirm that compounds C8 and C9 are the most potent dual A_1_/A_2A_ AR antagonists among our selected compounds.

### Novelty of the new hits

To evaluate the novelty of the identified dual A_1_/A_2A_ AR antagonists, the pairwise similarity between compounds C8 and C9 and known A_1_/A_2A_ AR antagonists, including all dual antagonists found in ChEMBL25 [53], was calculated using Morgan, ECFP4 and MACCS fingerprints in KNIME [54]. The molecular similarity between the two molecules was compared using the Tanimoto coefficient (Tc), which ranges from 0 to 1. Low chemical similarity has a correlation with a Tc value close to 0, whereas high chemical similarity has a correlation with a Tc value close to 1. Regarding compounds C8 and C9, the maximum Tc values of the Morgan and ECFP4 fingerprints compared to known A_1_/A_2A_ AR antagonists are similar and less than 0.4 (S3 Table). The maximum Tc values of MACCS and ECFP4 conform to the relationship studied by Maggiora et al.[55] (S6 Fig and S3 Table), indicating that compounds C8 and C9 are novel dual A_1_/A_2A_ AR antagonists. Compounds C8 and C9 contain a 1,2,4-triazole core, which is a privileged scaffold for developing potent antifungal agents [56], LSD1 inhibitors [57], anticonvulsant agents [58], etc. 1,2,4-Triazole derivatives possess diverse pharmacological activities, such as antiviral, antitumor, anti-inflammatory, antibacterial, antifungal, antihypertensive, hypoglycemic and analgesic activities [59]. The 1,2,4-triazole scaffold was first found to have the potential to be developed as a dual A_1_/A_2A_ AR antagonist. The associated pharmacological activity of compounds C8 and C9 acting as dual A_1_/A_2A_ AR antagonists needs to be further studied.

### Molecular modeling exploration

To explore the dual antagonistic activities of compound C8, C9 exhibited nanomolar inhibition against both A_1_AR and A_2A_AR, and molecular docking using Glide and MD simulation studies were carried out. In our docking studies, C8 and C9 had similar binding energies against A_1_AR and A_2A_AR (Table 5). In total, 4 complexes of C8 and C9 bound to A_1_AR and A_2A_AR were generated, and each complex was embedded in a hydrated phospholipid bilayer including POPE lipid molecules and subjected to 100-ns MD simulation using the AMBER ff14sb force field. After 100 ns of the MD simulation we obtained a stable bilayer (S7 Fig), and the RMS_prot_ (which is the protein backbone RMSD with respect to the minimized structure) values ranged from 2.51 to 2.96 Å with standard deviations of approximately 0.25 Å (S8 Fig). The stability of the ligand inside the binding area was assessed by measuring its RMSD (RMSD_lig_). Among the four docking poses used as starting structures, we obtained RMSD_lig_ less than 2 Å (Table 5 and S9 Fig), suggesting stable binding in agreement with the experimental binding and functional data.

**Table 5:** Glide XP scores, RMSD_lig_ and MM-GBSA binding free energies of ligands C8 and C9 against A_1_AR and A_2A_AR.

Compound ID	A_1_AR Glide XP (kcal/mol)	RMS_lig_ A_1_ (Å)	A_1_AR ΔG_eff_ (kcal/mol)	A_2A_AR Glide XP (kcal/mol)	RMS_lig_ A_2A_ (Å)	A_2A_AR ΔG_eff_ (kcal/mol)
C8	-11.36	1.69 ± 0.23	-41.67 ± 3.08	-10.50	0.92 ± 0.14	-42.22 ± 2.87
C9	-11.47	1.28 ± 0.24	-43.55 ± 2.62	-11.99	1.59 ± 0.19	-40.70 ± 2.83

In addition, we calculated the binding free energy of C8 and C9 with A_1_AR and A_2A_AR by using the MM-GBSA method based on the last 5 ns trajectory of the MD simulations (Table 5). The calculated binding free energy of the C8-A_1_AR complex (ΔG_eff_ = -41.67 kcal/mol) is similar to that of the C8-A_2A_AR complex (ΔG_eff_ = -42.22 kcal/mol). In addition, the calculated binding energy of the C9-A_1_AR complex (ΔG_eff_ = -43.55 kcal/mol) is similar to that of the C9-A_2A_AR complex (ΔG_eff_ = -40.70 kcal/mol). Therefore, the binding free energies show that compounds C8 and C9 have similar affinities to A_1_AR and A_2A_AR, which is consistent with the results of the functional assay and radioligand binding assays.

#### Binding modes exploration

**Fig 6:**
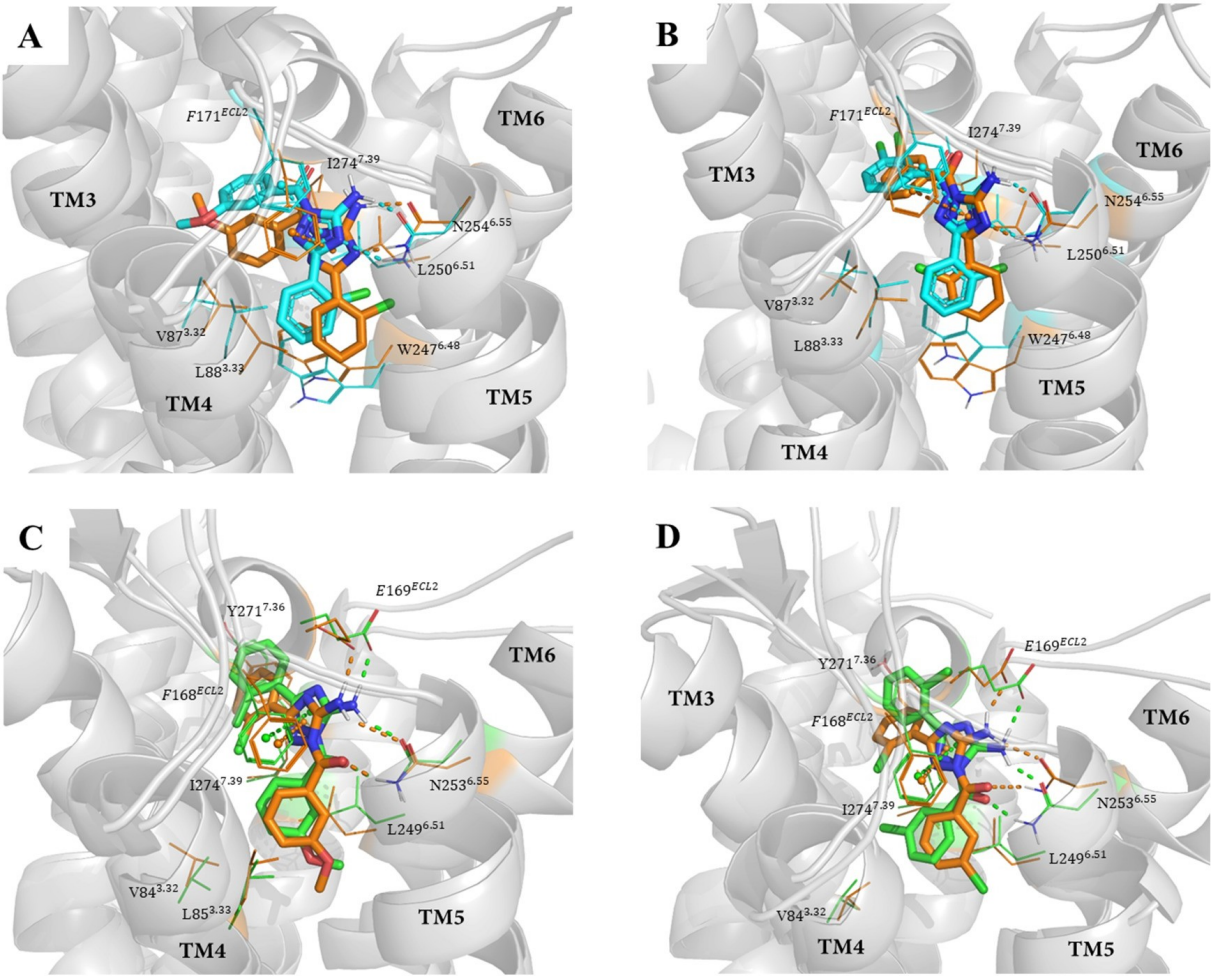
Superposition of the starting structure (in cyan) and the final snapshot (in orange) in the MD trajectory of Compounds C8 (A) and C9 (B) in the orthosteric binding area of A_1_AR. Superposition of the starting structure (in green) and the final snapshot (in orange) in the MD trajectory of Compounds C8 (C) and C9 (D) against A_2A_AR. The protein is shown as a gray cartoon, the ligands are shown as sticks, and the residues in the binding pocket of A_1_ and A_2A_ AR are represented as lines. The hydrogen bonds and π-π stacking between the ligands and ARs are represented by dashed lines.

The 100-ns MD simulations showed that compounds C8 and C9 were stabilized with similar orientations in the binding pockets of both A_1_AR and A_2A_AR (Fig 6). In the C8-A_1_AR complex and the C9-A_1_AR complex (Fig 6A and 6B), the 5-amino group and the nitrogen of the 1,2,4-triazol were hydrogen bonded to the side chain carbonyl and amino group of N254^6.55^, respectively. The 1,2,4-triazol formed π-π stacking interactions with the phenyl group of F171^ECL2^. These interactions are consistent with the observations in the mutagenesis experiments that N254^6.55^ and F171^ECL2^ play important roles in antagonist binding. These interactions were maintained from the origin to the end of the 100-ns simulation (S10 Fig). Additionally, the 3-methoxyphenyl (or 3-chorophenyl) exhibited nonpolar interactions with the side chain of I274^7.39^, and the 2-chorophenyl extended deeper into the other side of the pocket formed by the hydrophobic side chains of V87^3.32^, L88^3.33^ and W247^6.48^. In the C8-A_2A_AR complex and the C9-A_2A_AR complex (Fig 6C and 6D), the 5-amino group and the oxygen of the methanone formed hydrogen bonds with the side chain carbonyl and amino group of N253^6.55^. Additionally, the 5-amino group formed a hydrogen bond with the carboxyl side chain of E169^ECL2^. The 1,2,4-triazol interacted with the phenyl group of F168^ECL2^ through π-π stacking. These interactions were maintained throughout the 100-ns simulation (S11 Fig). The 2-chlorophenyl formed hydrophobic interactions with Y271^7.36^, and the 3-methoxyphenyl (or 3-chorophenyl) extended deeper into the orthosteric binding area and formed hydrophobic interactions with residues V84^3.32^, L85^3.33^, L249^6.51^ and I274^7.39^. Although the orientation of C8 (or C9) was different in the binding pocket of A_1_AR and A_2A_AR, the number of hydrogen bonds, π-π stacking and hydrophobic interactions were the same as those in the C8 (or C9)-A_1_AR complex and the C8 (or C9)-A_2A_AR complex, which may lead to the similar binding affinity of C8 (or C9) to A_1_AR and A_2A_AR.

**Fig 7:**
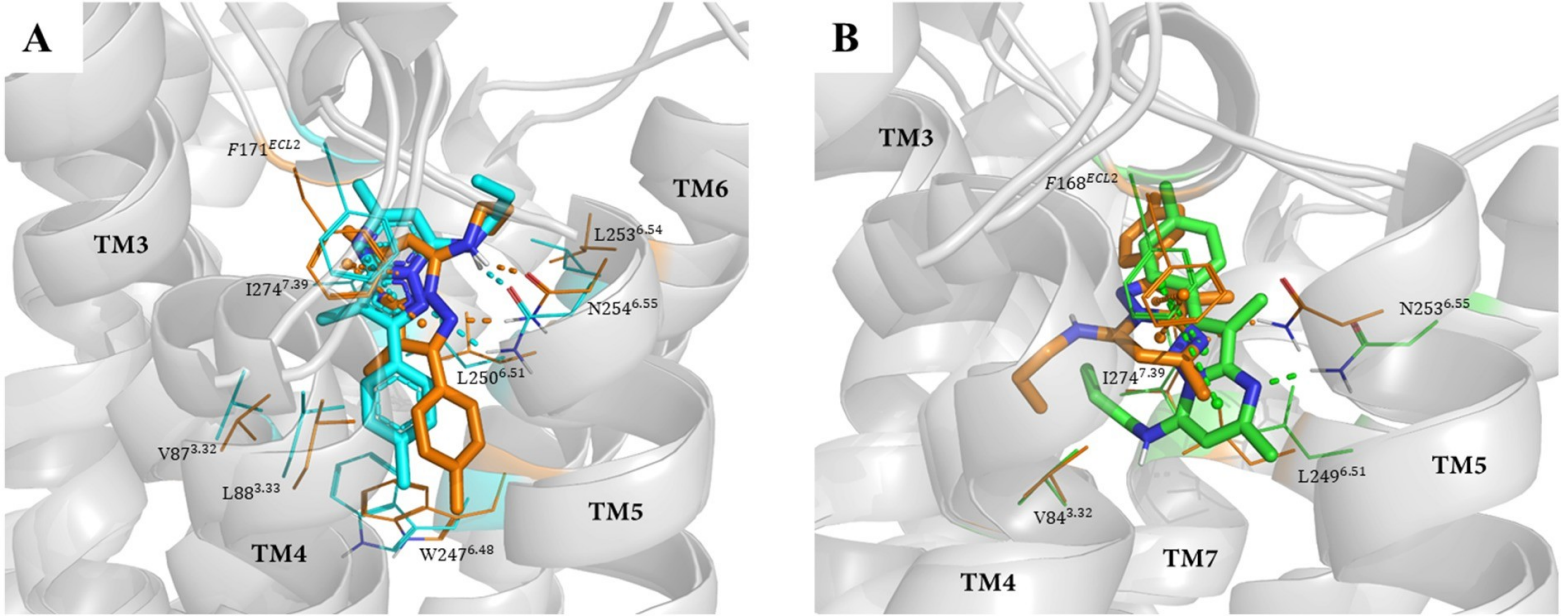
(A) Superposition of the starting structure (in cyan) and the last snapshot (in orange) in the MD trajectory of compound C10 in the orthosteric binding area of A_1_AR. (B) Superposition of the starting structure (in green) and the final snapshot (in orange) in the MD trajectory of compound C10 against A_2A_AR. The protein is shown as a gray cartoon, the ligands are shown as stick, and the residues in the binding pocket of A_1_ and A_2A_ AR are represented as lines. The hydrogen bonds and π-π stacking between compound C10 and ARs are represented by dashed lines.

To compare the binding differences between A_1_AR and A_2A_AR of nondual A_1_/A_2A_ AR antagonists, we also performed MD simulations against compound C10, which has stronger binding affinity and antagonistic activity to A_1_AR than A_2A_AR. In the C10-A_1_AR complex, the N-propyl group and the nitrogen of the pyrazolo[1,​5-​a]​pyrimidinformed two hydrogen bonds with the side chain carbonyl and amino group of N254^6.55^. Pyrazolo[1,​5-​a]​pyrimidin interacted with the phenyl side chain of F171^ECL2^ through π-π stacking. In the C10-A_2A_AR complex, C10 possessed a different orientation and formed only one hydrogen bond with the amino group of N253^6.55^ via the nitrogen of pyrazolo[1,​5-​a]​pyrimidin. Pyrazolo[1,​5-​a]​pyrimidin formed π-π stacking interactions with the phenyl side chain of F168^ECL2^ (Figs 7 and S12). Compared to the dual antagonists (which had similar a number of hydrogen bonds, π-π stacking and hydrophobic interactions in the two receptors), C10 had one less hydrogen bond in the C10-A_2A_AR complex than the C10-A_1_AR complex, which may be the reason for its weaker binding affinity and antagonistic activity for A_2A_AR.

## Conclusions

In the quest for novel dual A_1_/A_2A_ AR antagonists as putative agents for the treatment of Parkinson’s disease, we applied a multistage virtual screening approach combining deep learning, pharmacophore and molecular docking methods to screen the ChemDiv library (1,178,506 compounds) and tested 19 hits by *in vitro* assays. Initially, the cAMP functional assay identified five compounds that possessed antagonist activity towards A_1_/A_2A_AR with pIC_50_ values of 4.20–6.78. The radioligand binding assays confirmed that six of the eight compounds with antagonistic activity for A_1_AR and A_2A_AR (pIC_50_ of 4.20–6.78) also had consistent binding affinity against A_1_/A_2A_ ARs (pK_i_ of 4.71–7.49). In particular, compounds C8 and C9 showed the highest binding affinity and functional activity for A_1_/A_2A_ ARs with K_i_ values at the nanomolar level (pK_i_ of 7.16–7.49 and pIC_50_ of 6.31–6.78). Compounds C8 and C9 are novel 1,2,4-triazole derivatives acting as dual A_1_/A_2A_ AR antagonists. The MD simulations of the complexes between the A_1_/A_2A_ ARs and C8 and C9 further suggest strong binding interactions. Therefore, compounds C8 and C9 are hits with potential after optimization for the development of anti-Parkinson’s disease agents.

## Materials and methods

### Data set preparation

Dual antagonists with binding constants (K_i_) for A_1_AR and A_2A_AR subtypes were investigated in the ChEMBL25 database [53]. A dataset consisting of 310 bioactive dual antagonists from approximately 100 series (K_i_ < 40 nM) and 405 nonbioactive compounds in the same series (K_i_ > 1000 nM) were used to train the DNN and CNN classification models. The dataset was divided into a training set, test set and validation set at a ratio of 7:2:1.

Canvas similarity and clustering from Schrödinger were used to calculate the Tanimoto coefficients (Tc) of similarity between every pair of structures among 310 dual antagonists (K_i_ < 40 nM). Based on the calculated Tc values, similar structures were grouped together into the same cluster, yielding 14 clusters. Then, 14 representative structures of the diverse clusters were subsequently used to build dual antagonist pharmacophore models of the A_1_AR and A_2A_AR subtypes. To evaluate the performance of the pharmacophore models, an extra validation set comprising 42 dual antagonists (K_i_ < 40 nM) of A_1_AR and A_2A_AR subtypes and 913 decoy compounds was applied. With respect to known dual antagonists, the decoys were selected from the ZINC database using DecoyFinder [60] based on the following criteria: (1) Tanimoto coefficient < 0.75, (2) number of hydrogen bond acceptors ± 2, (3) number of hydrogen bond donors ± 1, (4) MW ± 25, (5) logP ± 1, and (6) Tanimoto coefficient less than 0.9 with respect to the other decoys.

All compounds in the training set, test set, validation set and ChemDiv library were prepared using Schrodinger’s Ligprep (version 10.2, Schrödinger, LLC) with the default settings to convert 2D structures to 3D structures, add hydrogen atoms, and generate tautomers, stereoisomers and protonation states at pH 7.0 ± 2.0 using Epik (version 4.3, Schrödinger, LLC).

### DNN and CNN

**Fig 8:**
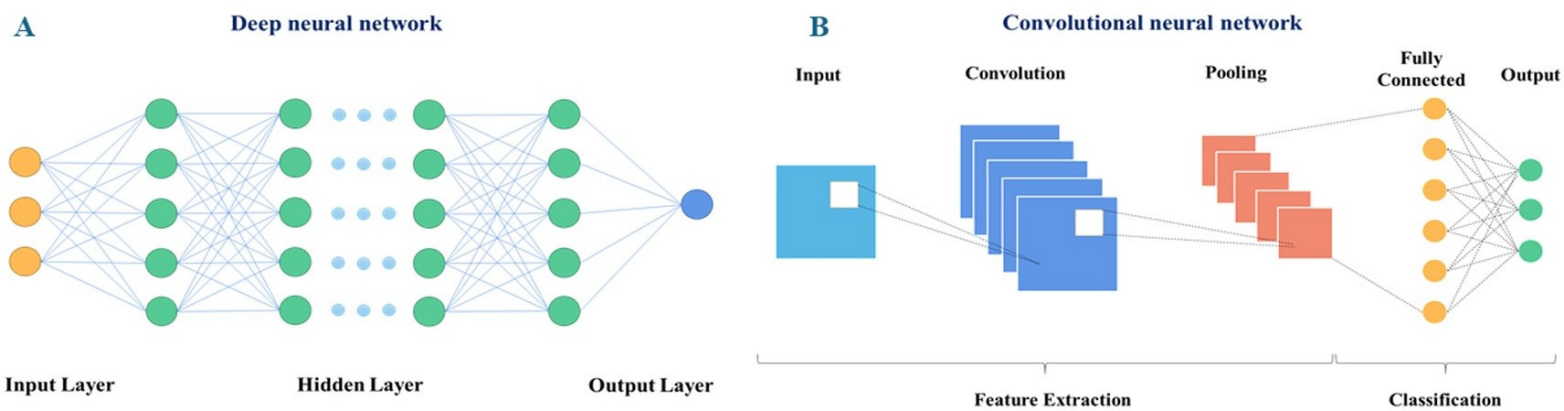
Architectures of neural networks. (A) Architecture of deep neural networks. (B) Architecture of convolutional neural networks.

A deep neural network (DNN) (Fig 8A) is a neural network with a certain complexity. DNNs comprise multiple levels of nonlinear operations and have many hidden layers. Neurons transfer information or signals to other neurons according to the received input, forming a complex network, and learning through some feedback mechanism [61]. A convolutional neural network (CNN) (Fig 8B) is a feed-forward neural network consisting of one or more convolutional layers and a fully connected layer at the top (corresponding to a classic neural network). CNNs also include associated weights and a pooling layer [62]. This structure enables the convolutional neural network to utilize the two-dimensional structure of the input data. Compared with other deep learning structures, convolutional neural networks can provide better results in image and speech recognition. Compared with other deep, feed-forward neural networks, convolutional neural networks need to consider fewer parameters, rendering these networks attractive deep learning structures.

Various statistical parameters were used to validate the performance of the DNN and CNN models. To evaluate the classification ability, parameters such as sensitivity (SE), specificity (SP), prediction accuracy of active molecules (Q+), prediction accuracy of inactive molecules (Q-), Matthews correlation coefficient (MCC) and the area under the receiver operating characteristic curve (AUC), were calculated using the following equations [63]:

$$SE=\frac{TP}{TP+FN}$$ (1)

$$SP=\frac{TN}{TN+FP}$$ (2)

$$Q+=\frac{TP}{TP+FP}$$ (3)

$$Q-=\frac{TN}{TN+FN}$$ (4)

$$MCC=\frac{TP*TN-FN*FP}{\sqrt{\left(TP+FN)(TP+FP)(TN+FN)(TN+FP\right)}}$$ (5)

where TP, FP, TN and FN denote true positives, false positives, true negatives and false negatives, respectively.

In this study, we aimed to improve the performance of dual A_1_/A_2A_ AR antagonist identification by deep learning. L2 regularization and “dropout” layers were used to prevent overfitting, and the rectified linear unit (ReLU) [64] was adopted as the activation function. The adaptive moment estimation (Adam) method [65] was used as an optimization algorithm for stochastic gradient descent in the process of training the deep learning models. In general, the performance of deep learning models is sensitive to the appropriate hyperparameters. To select the optimal hyperparameters for the deep learning models, a grid search approach [66] was used to optimize the hyperparameters based on the size of the training, including the learning rate, L2 regularization and number of hidden layers, and the size of the eigenvector in the hidden layers of the CNN model. In this stage, 209,585 and 171,233 compounds from the ChemDiv library (1,178,506 compounds) passed the filter that applied the DNN and CNN classification models, respectively. In total, 58,886 compounds that were simultaneously predicted by the DNN and CNN models were retained. The code and data sets used to train and test both the DNN and CNN models can be downloaded at https://github.com/Houshujing/Adenosine.

### Pharmacophore modeling

For the dual A_1_/A_2A_ AR antagonist pharmacophore generation, 14 diverse structures (K_i_ < 40 nM) selected from 310 dual antagonists were aligned in 3D space to generate common feature pharmacophores using the Phase module (version 5.4, Schrödinger, LLC). Each antagonist structure was minimized using the OPLS-2005 force field [67] implemented in Macromodel (version 11.9, Schrödinger, LLC), and described by a list of pharmacophore sites to characterize the chemical features contributing to the compound-protein interactions between the antagonists and A_1_AR and A_2A_AR subtypes. The perceived pharmacophore hypotheses were identified once the pharmacophore features were common to 50% of the antagonists. The generated pharmacophore hypotheses were examined using an extra validation set consisting of 42 dual antagonists and 913 decoy compounds. In this stage, 5,629 of the 58,886 compounds that matched the pharmacophore model were retained.

### Molecular docking

A Virtual Screening Workflow (version 7.8, Schrödinger, LLC) was used to perform the molecular docking calculations using the X-ray structures of A_1_AR cocrystallized with the antagonist PSB-36 (PDB ID 5N2S) and A_2A_AR cocrystallized with the antagonist ZM-241385 (PDB ID 5IU4). For each complex, the energy grid was generated at the centroid of the cocrystallized ligand. In addition, QikProp (version 5.5, Schrödinger, LLC) and Lipinski’s rule were applied to eliminate molecules with undesirable drug likeness properties. The 5,629 compounds that passed QikProp and Lipinski’s filters were docked and ranked sequentially using the Glide HTVS, SP and XP score functions based on the Glide score. The top 50% good scoring compounds through HTVS docking were filtered using SP. The top 25% good scoring compounds through SP docking were filtered using XP. Then, 43 of the top 25% good scoring compounds through XP against A_1_AR and A_2A_AR were further screened using visual inspection.

### Functional assay

In this work, 19 tested compounds were purchased from J&K Scientific Ltd. (Shanghai, China). Functional assays were performed by Pharmaron (Beijing) as previously described [52] by evaluating AR-mediated cAMP production using CHO-A_1_ cells and HEK293-A_2A_ cells stably expressing A_1_AR or A_2A_AR, respectively. The CHO-A_1_ cells and HEK293-A_2A_ cells were cultured in growth medium (Ham’s F12K (A_1_AR)/DMEM (A_2A_AR) + 10% FBS + 1*Ps + 400 μg/ml G418) at 37°C and 5% CO_2_, collected by centrifugation and resuspended in Hank’s balanced salt solution (HBSS), 0.1% bovine serum albumin (BSA), 20 mM N-(2-hydroxyethyl)-piperazine-N0-ethanesulfonic acid (HEPES) and 100 nM 3-isobutyl-1-methylxanthine (IBMX). The test compounds were serially diluted in DMSO at 3-fold dilutions, resulting in 10 concentrations starting from 5.08 × 10^−3^ to 100 μM. To evaluate the antagonist activity, the test compounds were added to the cell plate, and their ability to counteract the agonist (5’-N- ethylcarboxamidoadenosine, NECA)-mediated decrease in cAMP accumulation was assessed. cAMP production was determined using an ALPHAScreen cAMP kit according to the manufacturer’s instructions.

### Radioligand binding assays

The radioligand binding assays were also performed by Pharmaron (Beijing). In the A_1_AR and A_2A_AR assays, the test compounds were assessed in radioligand binding assays at human A_1_AR ([^3^H]DPCPX) and A_2A_AR ([^3^H]ZM241385). Displacement experiments of [^3^H]DPCPX (2.5 nM) against A_1_AR (4 μg/well) and ten different concentrations of test compounds were performed in 50 μL of assay buffer (50 mM Tris-HCl, 10 mM MgCl_2_, 1 mM EDTA, 1 μg/ml adenosine deaminase, pH 7.4) for 50 min at 25°C. The competition binding experiments of [^3^H]ZM241385 (0.5 nM) against A_2A_AR (4 μg/well) and ten different concentrations of the test compounds were performed in 500 μL of assay buffer (50 mM Tris-HCl, 10 mM MgCl_2_, 1 mM EDTA, 1 μg/ml adenosine deaminase, pH 7.4) for 90 min at 27°C.

Regarding the compounds that inhibited radioligand binding at A_1_AR and A_2A_AR by more than 50% at 100 μM, the IC_50_ values were determined from the sigmoidal concentration-response curves, which were generated using Xlfit (Version 5.3.1; IDBS, Guildford, UK). Depending on the IC_50_ values, the K_i_ values of the test compounds were calculated using the Cheng-Prusoff equation as follows: K_i_ = IC_50_/(1 + [C*]/K_d_*) [68]. In this equation, [C*] and K_d_* are the concentration of the radioligand and the equilibrium dissociation constant of the radioligand, respectively. The [C*]/K_d_* of the radioligands [^3^H]DPCPX and [^3^H]ZM241385 were 2.5 nM/2.1 nM and 0.5 nM/2.06 nM, respectively. S13 Fig shows the K_d_ determination curves of [^3^H]DPCPX and [^3^H]ZM241385.

### Molecular dynamics simulations

A_1_AR and A_2A_AR apo-proteins were extracted from the crystal structure of PSB36 or ZM241385 with thermally stable mutants A_1_AR or A_2A_AR (PDB ID: 5N2S and 5IU4) in the PDB database, respectively, and both apo-proteins and ligands were saved separately. The complex of PSB36 with thermostable mutant A_1_AR and the complex of ZM241385 with thermostable mutant A_2A_AR contain 9 and 10 mutation sites, respectively (5N2S: A54L^2.52^, T88A^3.36^, R107A^3.55^, K122A^4.43^, N154A^4.43^, L202A^5.63^, L235A^6.37^, V239A^6.41^, and S277A^7.42^; 5IU4: M1P^1.27^, A54L^2.52^, T88A^3.36^, R107A^3.55^, K122A^4.43^, N154A^ECL2^, L202A^5.63^, L235A^6.37^, V239A^6.41^, and S277A^7.42^). The mutant receptor residues were mutated back to the wild-type residues, the cocrystal T4 lysozyme was removed, and unnecessary small molecules were removed. The missing residues were added using the Prime module of Schrodinger. Hydrogen atoms were added through Schrodinger’s Protein Prepare module, and the protonation states of hydroxyl, Asn, Gln, and His in the structure were assigned by Schrodinger’s ProtAssign module [69].

PSB36 and ZM241385 were docked, and the resulting docking poses had RMSD values less than 1.8 Å from the experimental structure (S14 Fig and S4 Table). Then, we docked C8, C9 and C10 using the same methods and chose the docking pose with the lowest binding free energy. The 6 ligand-receptor complexes C8-A_1_AR, C8-A_2A_AR, C9-A_1_AR, C9-A_2A_AR, C10-A_1_AR and C10-A_2A_AR were used to perform the MD simulations. The transmembrane regions of A_1_AR and A_2A_AR were calculated through the Orientations of Proteins in Membranes (OPM) database [70]. The amino acid numbers of the seven transmembrane fragments of A_1_AR are 8–31, 46–71, 79–103, 124–144, 175–201, 236–259 and 267–287. The amino acid numbers of the seven transmembrane fragments of A_2A_AR are 3–29, 40–68, 78–101, 118–141, 174–200, 234–258 and 267–288. Each complex was inserted into a 95 Å× 80 Å POPE phospholipid bilayer through visualized operations in VMD [71], solvated using the TIP3P water model [72] and neutralized using 0.15 M NaCl. Finally, each system contains approximately 71,500 atoms, with approximately 140 lipids and approximately 16,500 waters, and the simulation cell had dimensions of 95 Å × 80 Å × 110 Å.

The MD simulations were carried out in triplicate using the PMEMD algorithm of AMBER 18 software [73]. The AMBER ff14SB force field [74] was used for the A_1_AR and A_2_AR systems. The standard protonation states of the protein residues were set at appropriate protonation states by the LEAP plugin in AMBER 18 as calculated using the H++ program [75]. The GAFF force field [76] and AMBER’s Antechamber were used to generate the topology and coordinate files of the compounds C8, C9 and C10. Lipid14 force field [77] was used for the POPE bilayer. First, each system was minimized by 10,000 steps. Second, a Langevin thermostat [78] was used to heat each system from 0 K to 310 K within 500 ps. Then, a 5-ns MD simulation in the NVT ensemble was performed while retaining the heavy atoms of the protein, ligand, and lipid head groups with a constraint of 50 kcal·mol^−1^·Å^−2^. Third, an MD simulation in the NPT ensemble was performed for 30 ns with a constraint for the protein and ligand. The restriction was gradually reduced from 50 (20 ns) to 10 (5 ns) and 2 kcal·mol^−1^· Å^−2^ (5 ns). Finally, each system was simulated for 100 ns without constraints under constant pressure (NPT ensemble) using a Berendsen barostat [79]. The cutoff value for the nonbonded interactions was set to 12 Å. The SHAKE algorithm [80] was used to constrain the covalent bonds involving hydrogen atoms, and the PME algorithm [81] was applied to address remote electrostatic interactions. In the process of the dynamics simulation, the time steps were set to 2 fs, and the frames were saved for analysis every 5,000 steps. The CPPTRAJ tool in AMBER 18 and VMD software [71] were used to analyze the trajectories.

The binding free energy (ΔG_eff_) [82,83] between the ligand and the protein was calculated using the MM-GBSA method [84–86] of the Python script MMPBSA.py [87]. For the calculation, the dielectric constant of the solvent was set to 80, and the dielectric constant of the solute (ℰ_in_) was set to 1 for the lipophilic binding area. The polar part of the free energy of desolvation (ΔG_GB_) was calculated using a modified GB model developed by Onufriev et al. [88]. The nonpolar part of the desolvation free energy (ΔG_SA_) was calculated based on the solvent accessible surface (SASA) prediction calculated by the LCPO algorithm [89].

## Supporting information

S1 Fig Matching graph of 19 compounds and a pharmacophore model (AAADR_1). (TIF)

S2 Fig (A)—(I): Concentration-response curves of compounds against A_1_AR in the cAMP assay. The data are presented as the mean ± SD of the inhibition percentage of cAMP production in duplicate assays.
(TIF)

S3 Fig (A)—(I): Concentration-response curves of compounds against A_2A_AR in the cAMP assay. The data are presented as the mean ± SD of the inhibition percentage of cAMP production in duplicate assays.
(TIF)

S4 Fig (A)—(I): Concentration-response curves of compounds against A_1_AR in the radioligand binding assay. The data are presented as the mean ± SD of the inhibition percentage of radioligand binding at A_1_AR in duplicate assays.
(TIF)

S5 Fig (A)—(I): Concentration-response curves of compounds against A_2A_AR in the radioligand binding assay. The data are presented as the mean ± SD of the inhibition percentage of radioligand binding at A_2A_AR in duplicate assays.
(TIF)

S6 Fig Corresponding Tc values of MACCS and ECFP4 [55]. Distributions of the Tc values of MACCS and ECFP4 were determined by conducting 10 million comparisons between randomly selected ZINC compounds. Correspondence between the Tc values of MACCS and ECFP4 was established by relating these Tc values to others that were met or exceeded by the same percentage of comparisons (indicated as labeled points on the curve).
(TIF)

S7 Fig Stable bilayer after 100 ns of MD simulations. (A) C8-A_1_AR complex embedded in the bilayer. (B) C9-A_1_AR complex embedded in the bilayer. (C) C8-A_2A_AR complex embedded in the bilayer. (D) C9-A_2A_AR complex embedded in the bilayer. The proteins are shown as blue (A_1_AR) and green (A_2A_AR) cartoons. The lipid molecules are represented as blue (A_1_AR) and green (A_2A_AR) lines. Sodium ions and chloride ions are represented as purple and green spheres. Water molecules are represented by red dots.
(TIF)

S8 Fig RMSDs of the protein in the C8-A_1_AR complex (A), C9-A_1_AR complex (B), C8-A_2A_AR complex (C) and C9-A_2A_AR complex (D) during the 100-ns MD simulations.
(TIF)

S9 Fig RMSDs of the ligand in the C8-A_1_AR complex (A), C9-A_1_AR complex (B), C8-A_2A_AR complex (C) and C9-A_2A_AR (D) during the 100-ns MD simulations.
(TIF)

S10 Fig N-N distance between 1,2,4-triazol and the side chain amino group of N254^6.55^ in the C8-A_1_AR complex (A) and C9-A_1_AR complex (D). N-O distance between the 5-amino group and the side chain carbonyl of N254^6.55^ in the C8-A_1_AR complex (B) and C9-A_1_AR complex (E). Distance between the centroids of 1,2,4-triazol and the side chain phenyl of F171^ECL2^ in the C8-A_1_AR complex (C) and C9-A_1_AR complex (F).
(TIF)

S11 Fig O-N distance between methanone and the side chain amino group of N253^6.55^ in the C8-A_2A_AR complex (A) and C9-A_2A_AR complex (E). N-O distance between the 5-amino group and the side chain carbonyl of N253^6.55^ in the C8-A_2A_AR complex (B) and C9-A_2A_AR complex (F). N-O distance between the 5-amino group and side chain of E169^ECL2^ in the C8-A_2A_AR complex (C) and C9-A_2A_AR complex (G). Distance between the centroids of 1,2,4-triazol and the side chain phenyl of F168^ECL2^ in the C8-A_2A_AR complex (D) and C9-A_2A_AR complex (H).
(TIF)

S12 Fig N-N distance between pyrazolo[1,5-a]pyrimidin and the side chain amino group of N254^6.55^ (or N253^6.55^) in the C10-A_1_AR complex (A) and C10-A_2A_AR complex (E). N-O distance between the N-propyl group and the side chain carbonyl of N254^6.55^ in the C10-A_1_AR complex (B). Distance between the centroids of pyrazole and the side chain phenyl of F171^ECL2^ (or F168^ECL2^) in the C10-A_1_AR complex (C) and C10-A_2A_AR complex (F). Distance between the centroids of pyrimidine and the side chain phenyl of F171^ECL2^ (or F168^ECL2^) in the C10-A_1_AR complex (D) and C10-A_2A_AR complex (G).
(TIF)

S13 Fig (A) Kd determination curves of [3H]DPCPX against A_1_AR in the filtration binding assay. Nonspecific signal: Different ligand concentrations of 10 μM DPCPX; (B) Kd determination curves of [3H]ZM241385 against A_2A_AR in the saturation binding assay. Nonspecific signal: Different ligand concentrations with 10 μM ZM241385. CPM = counts per minute, TB = total binding, NSB = nonspecific binding.
(TIF)

S14 Fig (A) Superposition of PSB36 in the orthosteric binding area of A_1_AR at the experimental structure (shown as sticks in white color) and the resulting docking pose (in green). (B) Superposition of ZM241385 in the orthosteric binding area of A_2A_AR at the experimental structure (shown as sticks in white color) and the resulting docking pose (in green). The protein is shown as a gray cartoon. The hydrogen bonds are represented by dashed lines. The side chains of F171, W247^6.48^, H251^6.52^, L253^6.54^, N254^6.55^, T257^6.58^ and H278^7.43^ (F168, N253^6.55^, L267^7.32^ and I274^7.39^ in A_2A_AR) are represented as lines.
(TIF)

S1 Table Training set for the dual A_1_/A_2A_ AR antagonist pharmacophore models. (PDF)

S2 Table Performance of various VS methods by screening the validation set with 433 dual A_1_/A_2A_ AR antagonists and 11,605 decoys. (PDF)

S3 Table Tc values of compounds C8 and C9 for Morgan, ECFP4 and MACCS. (PDF)

S4 Table RMSD between the redocked and crystallized conformations of PSB36 and ZM241385 by Glide XP. (PDF)

## References

[1] Kalia LV, Lang AE. Parkinson’s disease. The Lancet. 2015;386(9996):896–912. 10.1016/s0140-6736(14)61393-3 25904081

[2] Goetz CG. The history of Parkinson’s disease: early clinical descriptions and neurological therapies. Cold Spring Harb Perspect Med. 2011;1(1):a008862. 10.1101/cshperspect.a008862 22229124 PMC3234454

[3] Shook BC, Rassnick S, Osborne MC, Davis S, Westover L, Boulet J, et al. In vivo characterization of a dual adenosine A2A/A1 receptor antagonist in animal models of Parkinson’s disease. J Med Chem. 2010;53(22):8104–15. 10.1021/jm100971t . 20973483

[4] Chaudhuri KR, Yates L, Martinez-Martin P. The non-motor symptom complex of Parkinson’s disease: a comprehensive assessment is essential. Curr Neurol Neurosci Rep. 2005;5(4):275–83. 10.1007/s11910-005-0072-6 15987611

[5] Lacombe E, Carcenac C, Boulet S, Feuerstein C, Bertrand A, Poupard A, et al. High-frequency stimulation of the subthalamic nucleus prolongs the increase in striatal dopamine induced by acute l-3,4-dihydroxyphenylalanine in dopaminergic denervated rats. Eur J Neurosci. 2007;26(6):1670–80. Epub 2007/09/06. 10.1111/j.1460-9568.2007.05747.x . 17822436 PMC2798123

[6] Olanow CW, Agid Y, Mizuno Y, Albanese A, Bonuccelli U, Damier P, et al. Levodopa in the treatment of Parkinson’s disease: current controversies. Mov Disord. 2004;19(9):997–1005. 10.1002/mds.20243 . 15372588

[7] Mizuno Y. Definition and Classification of Parkinsonian Drugs. 2020. p. 1–30.

[8] Laurencin C, Danaila T, Broussolle E, Thobois S. Initial treatment of Parkinson’s disease in 2016: The 2000 consensus conference revisited. Revue Neurologique. 2016;172:512–23. 10.1016/j.neurol.2016.07.007 27476416

[9] Meissner WG, Frasier M, Gasser T, Goetz CG, Lozano A, Piccini P, et al. Priorities in Parkinson’s disease research. Nat Rev Drug Discov. 2011;10(5):377–93. 10.1038/nrd3430 . 21532567

[10] Lebon G, Warne T, Edwards PC, Bennett K, Langmead CJ, Leslie AG, et al. Agonist-bound adenosine A2A receptor structures reveal common features of GPCR activation. Nature. 2011;474(7352):521–5. 10.1038/nature10136 21593763 PMC3146096

[11] Carpenter B, Nehmé R, Warne T, Leslie AGW, Tate CG. Structure of the adenosine A2A receptor bound to an engineered G protein. Nature. 2016;536(7614):104–7. 10.1038/nature18966 http://www.nature.com/nature/journal/v536/n7614/abs/nature18966.html#supplementary-information. 27462812 PMC4979997

[12] Garcia-Nafria J, Lee Y, Bai X, Carpenter B, Tate CG. Cryo-EM structure of the adenosine A2A receptor coupled to an engineered heterotrimeric G protein. Elife. 2018;7. 10.7554/eLife.35946 29726815 PMC5962338

[13] Xu F, Wu H, Katritch V, Han GW, Jacobson KA, Gao ZG, et al. Structure of an agonist-bound human A2A adenosine receptor. Science. 2011;332(6027):322–7. 10.1126/science.1202793 21393508 PMC3086811

[14] Jaakola V-P, Griffith MT, Hanson MA, Cherezov V, Chien EY, Lane JR, et al. The 2.6 angstrom crystal structure of a human A2A adenosine receptor bound to an antagonist. Science. 2008;322(5905):1211–7. 10.1126/science.1164772 18832607 PMC2586971

[15] Liu W, Chun E, Thompson AA, Chubukov P, Xu F, Katritch V, et al. Structural Basis for Allosteric Regulation of GPCRs by Sodium Ions. Science. 2012;337(6091):232–6. 10.1126/science.1219218 22798613 PMC3399762

[16] Sun B, Bachhawat P, Chu ML-H, Wood M, Ceska T, Sands ZA, et al. Crystal structure of the adenosine A2A receptor bound to an antagonist reveals a potential allosteric pocket. Proc Natl Acad Sci. 2017;114(8):2066–71. 10.1073/pnas.1621423114 28167788 PMC5338372

[17] Dore AS, Robertson N, Errey JC, Ng I, Hollenstein K, Tehan B, et al. Structure of the adenosine A(2A) receptor in complex with ZM241385 and the xanthines XAC and caffeine. Structure. 2011;19(9):1283–93. 10.1016/j.str.2011.06.014 21885291 PMC3732996

[18] Glukhova A, Thal DM, Nguyen AT, Vecchio EA, Jörg M, Scammells PJ, et al. Structure of the adenosine A_1_ receptor reveals the basis for subtype selectivity. Cell. 2017;168(5):867–77. 10.1016/j.cell.2017.01.042 28235198

[19] Cheng RKY, Segala E, Robertson N, Deflorian F, Dore AS, Errey JC, et al. Structures of Human A1 and A2A Adenosine Receptors with Xanthines Reveal Determinants of Selectivity. Structure. 2017;25(8):1275–85 e4. 10.1016/j.str.2017.06.012 . 28712806

[20] Draper-Joyce CJ, Khoshouei M, Thal DM, Liang Y-L, Nguyen ATN, Furness SGB, et al. Structure of the adenosine-bound human adenosine A1 receptor–Gi complex. Nature. 2018;558(7711):559–63. 10.1038/s41586-018-0236-6 29925945

[21] Katritch V, Jaakola VP, Lane JR, Lin J, Ijzerman AP, Yeager M, et al. Structure-based discovery of novel chemotypes for adenosine A(2A) receptor antagonists. J Med Chem. 2010;53(4):1799–809. 10.1021/jm901647p 20095623 PMC2826142

[22] Carlsson J, Yoo L, Gao ZG, Irwin JJ, Shoichet BK, Jacobson KA. Structure-based discovery of A2A adenosine receptor ligands. J Med Chem. 2010;53(9):3748–55. 10.1021/jm100240h 20405927 PMC2865168

[23] Lagarias P, Vrontaki E, Lambrinidis G, Stamatis D, Convertino M, Ortore G, et al. Discovery of Novel Adenosine Receptor Antagonists through a Combined Structure- and Ligand-Based Approach Followed by Molecular Dynamics Investigation of Ligand Binding Mode. J Chem Inf Model. 2018;58(4):794–815. 10.1021/acs.jcim.7b00455 . 29485875

[24] Fredholm BB, AP IJ, Jacobson KA, Linden J, Muller CE. International Union of Basic and Clinical Pharmacology. LXXXI. Nomenclature and classification of adenosine receptors—an update. Pharmacol Rev. 2011;63(1):1–34. 10.1124/pr.110.003285 21303899 PMC3061413

[25] Yacoubi ME, Ledent C, Parmentier M, Bertorelli R, Ongini E, Costentin J, et al. Adenosine A2A receptor antagonists are potential antidepressants: evidence based on pharmacology and A2A receptor knockout mice. Br J Pharmacol. 2001;134(1):68–77. 10.1038/sj.bjp.0704240 11522598 PMC1572930

[26] Yamada K, Kobayashi M, Mori A, Jenner P, Kanda T. Antidepressant-like activity of the adenosine A(2A) receptor antagonist, istradefylline (KW-6002), in the forced swim test and the tail suspension test in rodents. Pharmacol Biochem Behav. 2013;114–115:23–30. 10.1016/j.pbb.2013.10.022 . 24201052

[27] Yamada K, Kobayashi M, Shiozaki S, Ohta T, Mori A, Jenner P, et al. Antidepressant activity of the adenosine A2A receptor antagonist, istradefylline (KW-6002) on learned helplessness in rats. Psychopharmacology (Berl). 2014;231(14):2839–49. 10.1007/s00213-014-3454-0 . 24488405

[28] Chen J-F, Xu K, Petzer JP, Staal R, Xu Y-H, Beilstein M, et al. Neuroprotection by caffeine and A2A adenosine receptor inactivation in a model of Parkinson’s disease. J Neurosci. 2001;21(10):RC143–RC. 10.1523/JNEUROSCI.21-10-j0001.2001 11319241 PMC6762498

[29] van Rensburg HJ, Legoabe L, Terre’Blanche G, Van der Walt M. 2–Benzylidene–1–Indanone Analogues as Dual Adenosine A1/A2a Receptor Antagonists for the Potential Treatment of Neurological Conditions. Drug Res. 2019;69(07):382–91. 10.1055/a-0808-3993 30616250

[30] Bortolotto JW, de Melo GM, de Paula Cognato G, Vianna MRM, Bonan CD. Modulation of adenosine signaling prevents scopolamine-induced cognitive impairment in zebrafish. Neurobiol Learn Mem. 2015;118:113–9. 10.1016/j.nlm.2014.11.016 25490060

[31] Ross GW, Abbott RD, Petrovitch H, Morens DM, Grandinetti A, Tung K-H, et al. Association of coffee and caffeine intake with the risk of Parkinson disease. JAMA. 2000;283(20):2674–9. 10.1001/jama.283.20.2674 10819950

[32] Shook BC, Rassnick S, Chakravarty D, Wallace N, Ault M, Crooke J, et al. Optimization of arylindenopyrimidines as potent adenosine A(2A)/A(1) antagonists. Bioorg Med Chem Lett. 2010;20(9):2868–71. 10.1016/j.bmcl.2010.03.024 . 20338760

[33] Atack JR, Shook BC, Rassnick S, Jackson PF, Rhodes K, Drinkenburg WH, et al. JNJ-40255293, a novel adenosine A2A/A1 antagonist with efficacy in preclinical models of Parkinson’s disease. ACS Chem Neurosci. 2014;5(10):1005–19. 10.1021/cn5001606 . 25203719

[34] Shook BC, Jackson PF. Adenosine A(2A) Receptor Antagonists and Parkinson’s Disease. ACS Chem Neurosci. 2011;2(10):555–67. 10.1021/cn2000537 22860156 PMC3369712

[35] Shook BC, Rassnick S, Wallace N, Crooke J, Ault M, Chakravarty D, et al. Design and characterization of optimized adenosine A(2)A/A(1) receptor antagonists for the treatment of Parkinson’s disease. J Med Chem. 2012;55(3):1402–17. 10.1021/jm201640m . 22239465

[36] Mihara T, Mihara K, Yarimizu J, Mitani Y, Matsuda R, Yamamoto H, et al. Pharmacological characterization of a novel, potent adenosine A1 and A2A receptor dual antagonist, 5-[5-amino-3-(4-fluorophenyl)pyrazin-2-yl]-1-isopropylpyridine-2(1H)-one (ASP5854), in models of Parkinson’s disease and cognition. J Pharmacol Exp Ther. 2007;323(2):708–19. 10.1124/jpet.107.121962 . 17684118

[37] Mihara T, Iwashita A, Matsuoka N. A novel adenosine A(1) and A(2A) receptor antagonist ASP5854 ameliorates motor impairment in MPTP-treated marmosets: comparison with existing anti-Parkinson’s disease drugs. Behav Brain Res. 2008;194(2):152–61. 10.1016/j.bbr.2008.06.035 . 18657577

[38] Robinson SJ, Petzer JP, Terre’Blanche G, Petzer A, van der Walt MM, Bergh JJ, et al. 2-Aminopyrimidines as dual adenosine A1/A2A antagonists. Eur J Med Chem. 2015;104:177–88. 10.1016/j.ejmech.2015.09.035 . 26462195

[39] Geldenhuys WJ, Hanif A, Yun J, Nayeem MA. Exploring Adenosine Receptor Ligands: Potential Role in the Treatment of Cardiovascular Diseases. Molecules. 2017;22(6). 10.3390/molecules22060917 28587166 PMC5568125

[40] Antonioli L, Csoka B, Fornai M, Colucci R, Kokai E, Blandizzi C, et al. Adenosine and inflammation: what’s new on the horizon? Drug Discov Today. 2014;19(8):1051–68. 10.1016/j.drudis.2014.02.010 . 24607729

[41] Kristy A, Carpenter, David S, Cohen, Juliet T, Jarrell, et al. Deep learning and virtual drug screening. Future medicinal chemistry. 2018;10(21):2557–67. 10.4155/fmc-2018-0314 30288997 PMC6563286

[42] Unterthiner T, Mayr A, Klambauer G, Steijaert M, Hochreiter S, editors. Deep Learning as an Opportunity in Virtual Screening. Workshop on Deep Learning and Representation Learning (NIPS2014); 2014.

[43] Lenselink EB, ten Dijke N, Bongers B, Papadatos G, van Vlijmen HWT, Kowalczyk W, et al. Beyond the hype: deep neural networks outperform established methods using a ChEMBL bioactivity benchmark set. Journal of Cheminformatics. 2017;9(1):45. 10.1186/s13321-017-0232-0 29086168 PMC5555960

[44] Bilsland AE, Pugliese A, Liu Y, Revie J, Burns S, McCormick C, et al. Identification of a Selective G1-Phase Benzimidazolone Inhibitor by a Senescence-Targeted Virtual Screen Using Artificial Neural Networks. Neoplasia. 2015;17(9):704–15. 10.1016/j.neo.2015.08.009 . 26476078 PMC4611071

[45] Wallach I, Dzamba M, Heifets A. AtomNet: A Deep Convolutional Neural Network for Bioactivity Prediction in Structure-based Drug Discovery. Computer Science 2015.

[46] Rifaioglu AS, Atalay V, Martin MJ, Cetin-Atalay R, Science TJC. DEEPScreen: High Performance Drug-Target Interaction Prediction with Convolutional Neural Networks Using 2-D Structural Compound Representations. Chemical Science 2020;11:2531–57. 10.1039/c9sc03414e 33209251 PMC7643205

[47] Schaller D, Šribar D, Noonan T, Deng L, Nguyen TN, Pach S, et al. Next generation 3D pharmacophore modeling. WIREs Computational Molecular Science. 2020;10(4). 10.1002/wcms.1468

[48] Lyu J, Wang S, Balius TE, Singh I, Levit A, Moroz YS, et al. Ultra-large library docking for discovering new chemotypes. Nature. 2019;566(7743):224–9. 10.1038/s41586-019-0917-9 30728502 PMC6383769

[49] Rogers D, Hahn M. Extended-connectivity fingerprints. Journal of Chemical Information Modeling. 2010;50(5):742–54. 10.1021/ci100050t 20426451

[50] Duvenaud D, Maclaurin D, Aguilera-Iparraguirre J, Gómez-Bombarelli R, Hirzel T, Aspuru-Guzik A, et al. Convolutional Networks on Graphs for Learning Molecular Fingerprints. Proceedings of the 28th International Conference on Neural Information Processing Systems. 2015;2:2224–32.

[51] Truchon J-Fo Bayly CI. Evaluating Virtual Screening Methods:? Good and Bad Metrics for the "Early Recognition" Problem. journal of chemical information and modeling. 2007;47(2):488–508. 10.1021/ci600426e 17288412

[52] Wei Y, Wang M, Li Y, Hong Z, Li D, Lin J. Identification of new potent A1 adenosine receptor antagonists using a multistage virtual screening approach. Eur J Med Chem. 2019;187:111936. 10.1016/j.ejmech.2019.111936 . 31855793

[53] Mendez D, Gaulton A, Bento AP, Chambers J, Veij MD, Félix E, et al. ChEMBL: towards direct deposition of bioassay data. Nucleic Acids Research. 2019;47:D930–D40. 10.1093/nar/gky1075 30398643 PMC6323927

[54] Berthold M, Cebron N, Dill F, Gabriel T, Kötter T, Meinl T, et al. KNIME—the Konstanz information miner. ACM SIGKDD Explorations Newsletter. 2009;11:26–31. 10.1145/1656274.1656280

[55] Maggiora G, Vogt M, Stumpfe D, Bajorath J. Molecular similarity in medicinal chemistry. J Med Chem. 2014;57(8):3186–204. 10.1021/jm401411z . 24151987

[56] Tratrat C. 1,2,4-Triazole A Privileged Scaffold For The Development Of Potent Antifungal Agents-A Brief Review. Current Topics in Medicinal Chemistry. 2020;7(4). 10.2174/1568026620666200704140107 32621720

[57] Kutz CJ, Holshouser SL, Marrow EA, Woster PMJM. 3,5-Diamino-1,2,4-triazoles as a novel scaffold for potent, reversible LSD1 (KDM1A) inhibitors. Medchemcomm. 2014;5(12):1863–70. 10.1039/C4MD00283K 25580204 PMC4286191

[58] Vk K, Pk V, A D, Ranjan S. 1,2,4-triazole derivatives as potential scaffold for anticonvulsant activity. Central Nervous System Agents in Medicinal Chemistry. 2015;15(1):17–22. 10.2174/1871524915666150209100533 25675400

[59] Thakur A, Shukla P, Verma A, Pathak P. 1, 2, 4-Triazole Scafolds: Recent Advances and Pharmacological Applications. International Journal of Current Research and Academic Review. 2016;4:277–96. 10.20546/ijcrar.2016.402.031

[60] Cereto-Massagué A, Guasch L, Valls C, Mulero M, Pujadas G, Garcia-Vallve S. DecoyFinder: An easy-to-use python GUI application for building target-specific decoy sets. Bioinformatics (Oxford, England). 2012;28:1661–2. 10.1093/bioinformatics/bts249 22539671

[61] Kriegeskorte N. Deep Neural Networks: A New Framework for Modeling Biological Vision and Brain Information Processing. Annu Rev Vis Sci. 2015;1:417–46. 10.1146/annurev-vision-082114-035447 . 28532370

[62] Valueva MV, Nagornov NN, Lyakhov PA, Valuev GV, Chervyakov NI. Application of the residue number system to reduce hardware costs of the convolutional neural network implementation. Mathematics and Computers in Simulation. 2020;177:232–43. 10.1016/j.matcom.2020.04.031

[63] Jamal S, Scaria V. Cheminformatic models based on machine learning for pyruvate kinase inhibitors of Leishmania Mexicana. BMC Bioinformatics. 2013;14(1):329. 10.1186/1471-2105-14-329 24252103 PMC4225525

[64] Nair V, Hinton G. Rectified Linear Units Improve Restricted Boltzmann Machines Vinod Nair 2010. 807–14 p.

[65] Kingma D, Ba J. Adam: A Method for Stochastic Optimization. International Conference on Learning Representations. 2014.

[66] Hinton G. A Practical Guide to Training Restricted Boltzmann Machines. Momentum. 2012;9(1):599–619.

[67] Kaminski GA, Friesner RA, Tirado-Rives J, Jorgensen WL. Evaluation and reparametrization of the OPLS-AA force field for proteins via comparison with accurate quantum chemical calculations on peptides. The Journal of Physical Chemistry B. 2001;105(28):6474–87.

[68] Yung-Chi C, Prusoff W. Relationship Between the Inhibition Constant (KI) and the Concentration of Inhibitor Which Causes 50 Per Cent Inhibition (I50) of an Enzymatic Reaction. Biochemical Pharmacology. 1973;22:3099–108. 10.1016/0006-2952(73)90196-2 4202581

[69] Sastry GM, Adzhigirey M, Day T, Annabhimoju R, Sherman W. Protein and ligand preparation: parameters, protocols, and influence on virtual screening enrichments. J Comput-Aided Mol Des. 2013;27(3):221–34. 10.1007/s10822-013-9644-8 23579614

[70] Lomize MA, Pogozheva ID, Joo H, Mosberg HI, Lomize AL. OPM database and PPM web server: resources for positioning of proteins in membranes. Nucleic Acids Res. 2012;40(D1):D370–D6. 10.1093/nar/gkr703 21890895 PMC3245162

[71] Humphrey W, Dalke A, Schulten K. VMD: visual molecular dynamics. J Mol Graph. 1996;14(1):33–8. 10.1016/0263-7855(96)00018-5 8744570

[72] Jorgensen WL, Chandrasekhar J, Madura JD, Impey RW, Klein ML. Comparison of simple potential functions for simulating liquid water. J Chem Phys. 1983;79(2):926–35.

[73] Katritch V, Cherezov V, Stevens RC. Structure-function of the G protein-coupled receptor superfamily. Annu Rev Pharmacool Toxicol. 2013;53(1):531–56. 10.1146/annurev-pharmtox-032112-135923 . 23140243 PMC3540149

[74] Maier JA, Martinez C, Kasavajhala K, Wickstrom L, Hauser KE, Simmerling C. ff14SB: improving the accuracy of protein side chain and backbone parameters from ff99SB. J Chem Theory Comput. 2015;11(8):3696–713. 10.1021/acs.jctc.5b00255 26574453 PMC4821407

[75] Anandakrishnan R, Aguilar B, Onufriev AV. H++ 3.0: automating pK prediction and the preparation of biomolecular structures for atomistic molecular modeling and simulations. Nucleic Acids Res. 2012;40(Web Server issue):W537–41. 10.1093/nar/gks375 22570416 PMC3394296

[76] Wang J, Wang W, Kollman PA, Case DA. Automatic atom type and bond type perception in molecular mechanical calculations. J Mol Graphics Modell. 2006;25(2):247–60. 10.1016/j.jmgm.2005.12.005 16458552

[77] Dickson CJ, Madej BD, Skjevik ÅA, Betz RM, Teigen K, Gould IR, et al. Lipid14: the amber lipid force field. J Chem Theory Comput. 2014;10(2):865–79. 10.1021/ct4010307 24803855 PMC3985482

[78] Pastor RW, Brooks BR, Szabo A. An analysis of the accuracy of Langevin and molecular dynamics algorithms. Mol Phys. 1988;65(6):1409–19.

[79] Berendsen HJ, Postma Jv, van Gunsteren WF, DiNola A, Haak JR. Molecular dynamics with coupling to an external bath. J Chem Phys. 1984;81(8):3684–90.

[80] Ryckaert J-P, Ciccotti G, Berendsen HJ. Numerical integration of the cartesian equations of motion of a system with constraints: molecular dynamics of n-alkanes. J Comput Phys. 1977;23(3):327–41.

[81] Darden T, York D, Pedersen L. Particle mesh Ewald: An N· log (N) method for Ewald sums in large systems. J Chem Phys. 1993;98(12):10089–92.

[82] Genheden S, Ryde U. The MM/PBSA and MM/GBSA methods to estimate ligand-binding affinities. Expert opinion on drug discovery. 2015;10(5):449–61. 10.1517/17460441.2015.1032936 25835573 PMC4487606

[83] Miller BR III, McGee TD Jr, Swails JM, Homeyer N, Gohlke H, Roitberg AE. MMPBSA. py: an efficient program for end-state free energy calculations. J Chem Theory Comput. 2012;8(9):3314–21. 10.1021/ct300418h 26605738

[84] Tsui V, Case DA. Theory and applications of the generalized Born solvation model in macromolecular simulations. Biopolymers: Original Research on Biomolecules. 2000;56(4):275–91. 10.1002/1097-0282(2000)56:4&lt;275::AID-BIP10024&gt;3.0.CO;2-E 11754341

[85] Kollman PA, Massova I, Reyes C, Kuhn B, Huo S, Chong L, et al. Calculating structures and free energies of complex molecules: combining molecular mechanics and continuum models. Acc Chem Res. 2000;33(12):889–97. 10.1021/ar000033j 11123888

[86] Peddi SR, Sivan SK, Manga V. Molecular dynamics and MM/GBSA-integrated protocol probing the correlation between biological activities and binding free energies of HIV-1 TAR RNA inhibitors. J Biomol Struct Dyn. 2018;36(2):486–503. 10.1080/07391102.2017.1281762 28081678

[87] Miller BR 3rd, McGee TD Jr., Swails JM, Homeyer N, Gohlke H, Roitberg AE. MMPBSA.py: An Efficient Program for End-State Free Energy Calculations. J Chem Theory Comput. 2012;8(9):3314–21. 10.1021/ct300418h . 26605738

[88] Onufriev A, Bashford D, Case DA. Modification of the generalized Born model suitable for macromolecules. The Journal of Physical Chemistry B. 2000;104(15):3712–20.

[89] Weiser J, Shenkin PS, Still WC. Approximate atomic surfaces from linear combinations of pairwise overlaps (LCPO). J Comput Chem. 1999;20(2):217–30.

