# Supplementary figures and images for: Discovery of novel dual adenosine A1/A2A receptor antagonists using deep learning, pharmacophore modeling and molecular docking
Source: PLoS Comput Biol. 2021 Mar 19;17(3):e1008821. doi: 10.1371/journal.pcbi.1008821 (PMC7978378; doi:10.1371/journal.pcbi.1008821)

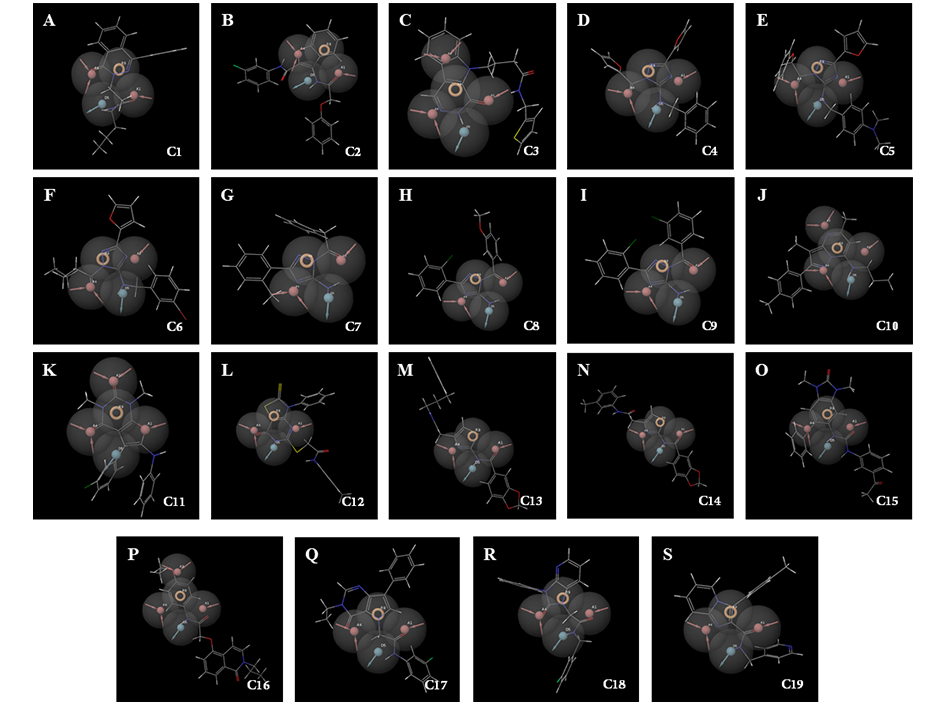

Supplement: S1 Fig — (TIF) [file pcbi.1008821.s001.tif]

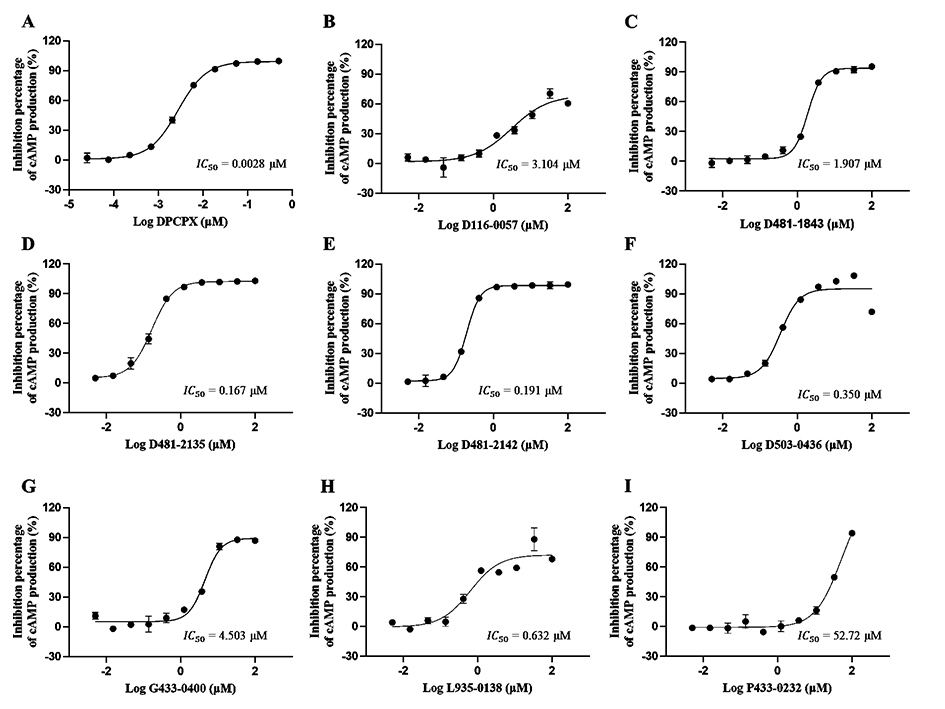

Supplement: S2 Fig — (A)—(I): Concentration-response curves of compounds against A1AR in the cAMP assay. The data are presented as the mean ± SD of the inhibition percentage of cAMP production in duplicate assays. (TIF) [file pcbi.1008821.s002.tif]

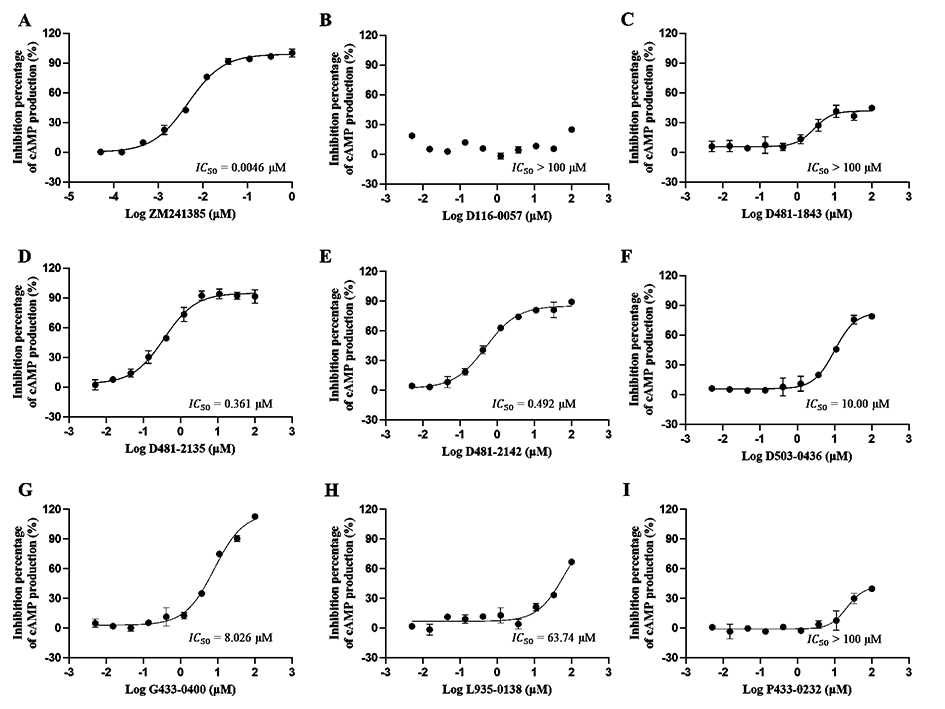

Supplement: S3 Fig — (A)—(I): Concentration-response curves of compounds against A2AAR in the cAMP assay. The data are presented as the mean ± SD of the inhibition percentage of cAMP production in duplicate assays. (TIF) [file pcbi.1008821.s003.tif]

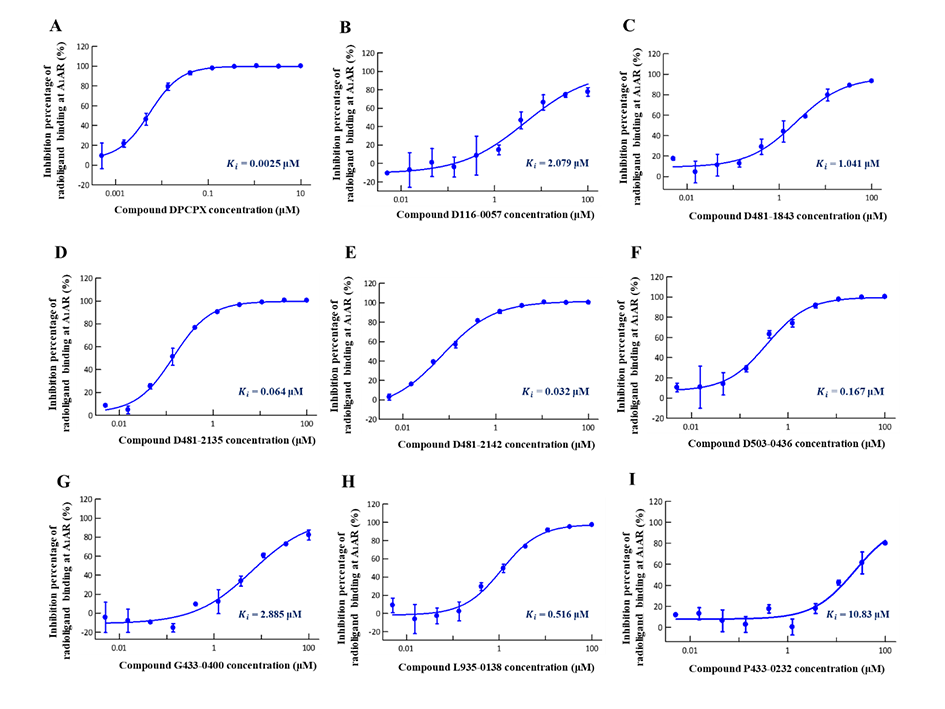

Supplement: S4 Fig — (A)—(I): Concentration-response curves of compounds against A1AR in the radioligand binding assay. The data are presented as the mean ± SD of the inhibition percentage of radioligand binding at A1AR in duplicate assays. (TIF) [file pcbi.1008821.s004.tif]

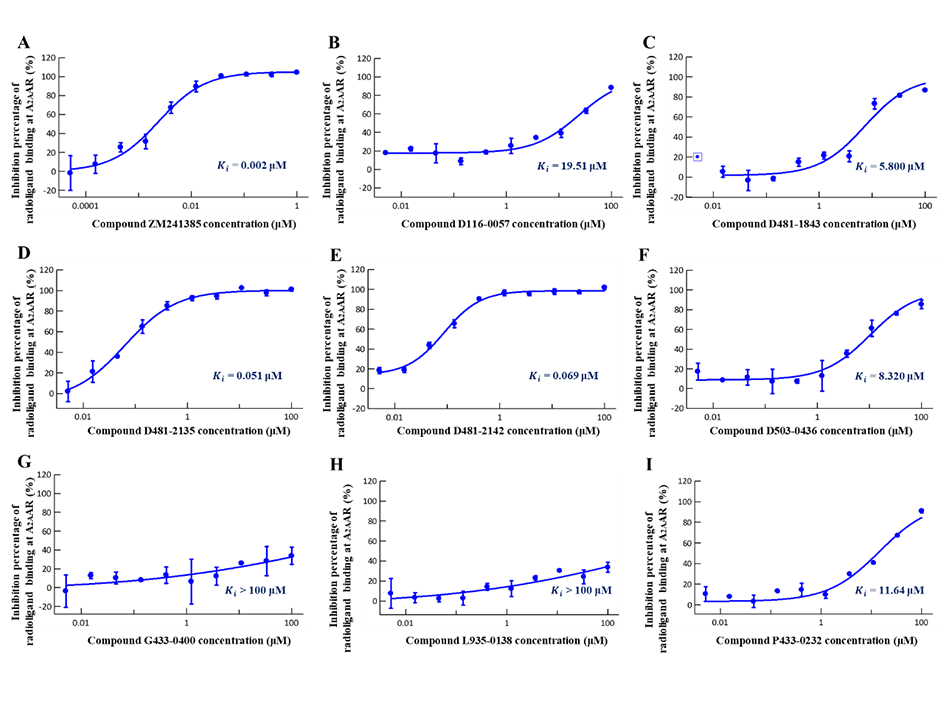

Supplement: S5 Fig — (A)—(I): Concentration-response curves of compounds against A2AAR in the radioligand binding assay. The data are presented as the mean ± SD of the inhibition percentage of radioligand binding at A2AAR in duplicate assays. (TIF) [file pcbi.1008821.s005.tif]

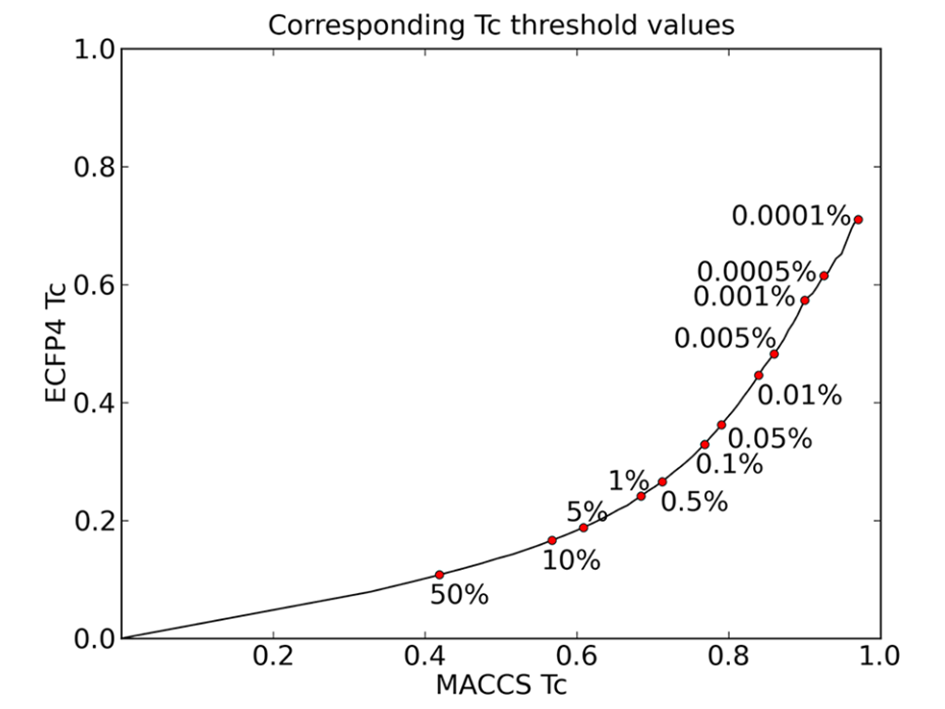

Supplement: S6 Fig — Distributions of the Tc values of MACCS and ECFP4 were determined by conducting 10 million comparisons between randomly selected ZINC compounds. Correspondence between the Tc values of MACCS and ECFP4 was established by relating these Tc values to others that were met or exceeded by the same percentage of comparisons (indicated as labeled points on the curve). (TIF) [file pcbi.1008821.s006.tif]

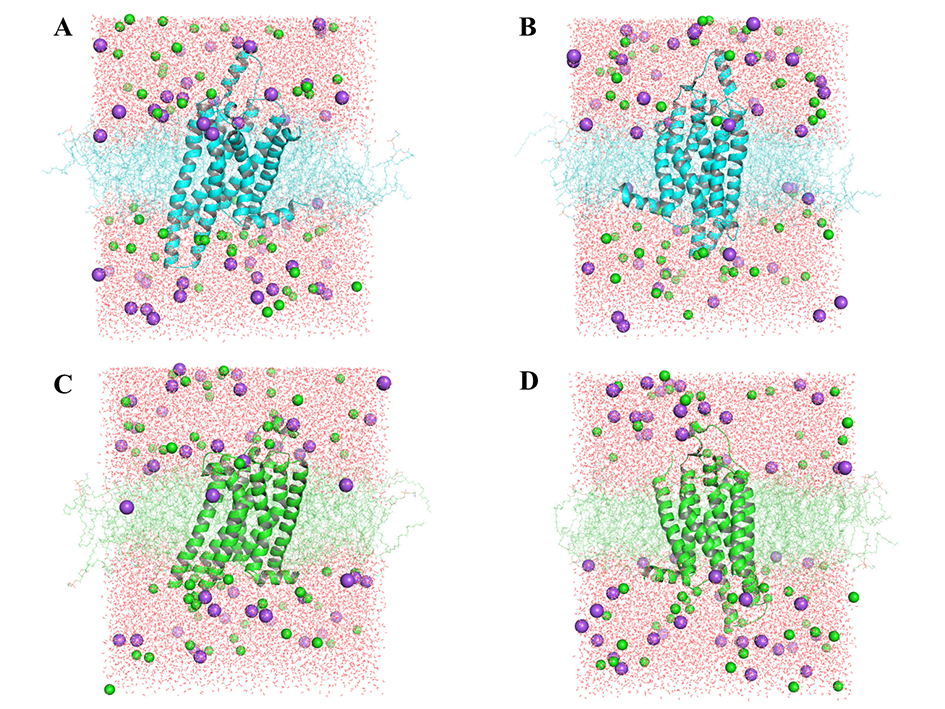

Supplement: S7 Fig — (A) C8-A1AR complex embedded in the bilayer. (B) C9-A1AR complex embedded in the bilayer. (C) C8-A2AAR complex embedded in the bilayer. (D) C9-A2AAR complex embedded in the bilayer. The proteins are shown as blue (A1AR) and green (A2AAR) cartoons. The lipid molecules are represented as blue (A1AR) and green (A2AAR) lines. Sodium ions and chloride ions are represented as purple and green spheres. Water molecules are represented by red dots. (TIF) [file pcbi.1008821.s007.tif]

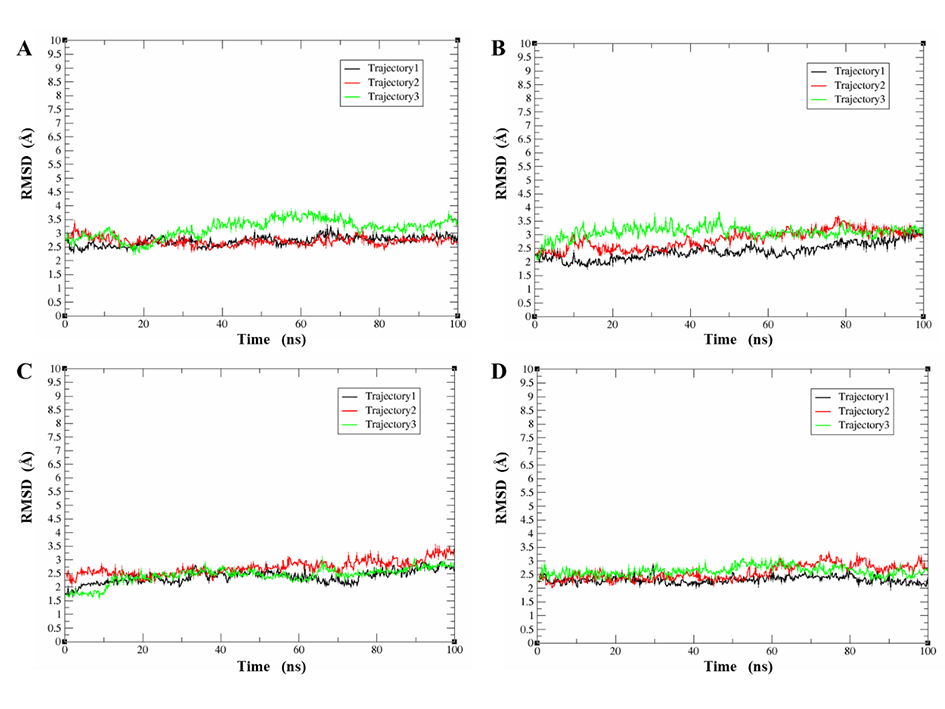

Supplement: S8 Fig — RMSDs of the protein in the C8-A1AR complex (A), C9-A1AR complex (B), C8-A2AAR complex (C) and C9-A2AAR complex (D) during the 100-ns MD simulations. (TIF) [file pcbi.1008821.s008.tif]

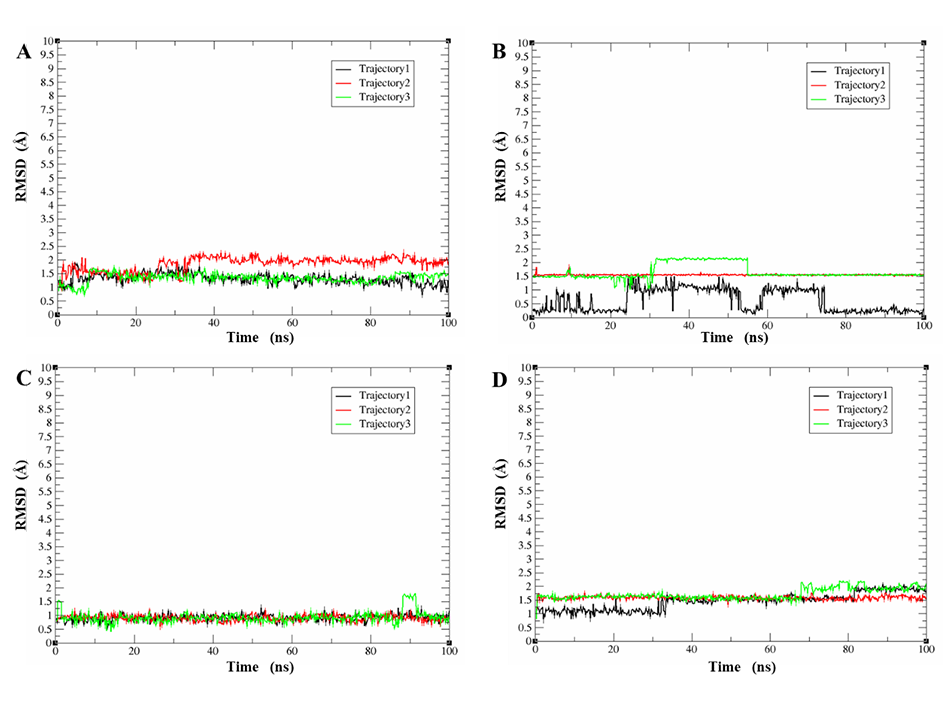

Supplement: S9 Fig — RMSDs of the ligand in the C8-A1AR complex (A), C9-A1AR complex (B), C8-A2AAR complex (C) and C9-A2AAR (D) during the 100-ns MD simulations. (TIF) [file pcbi.1008821.s009.tif]

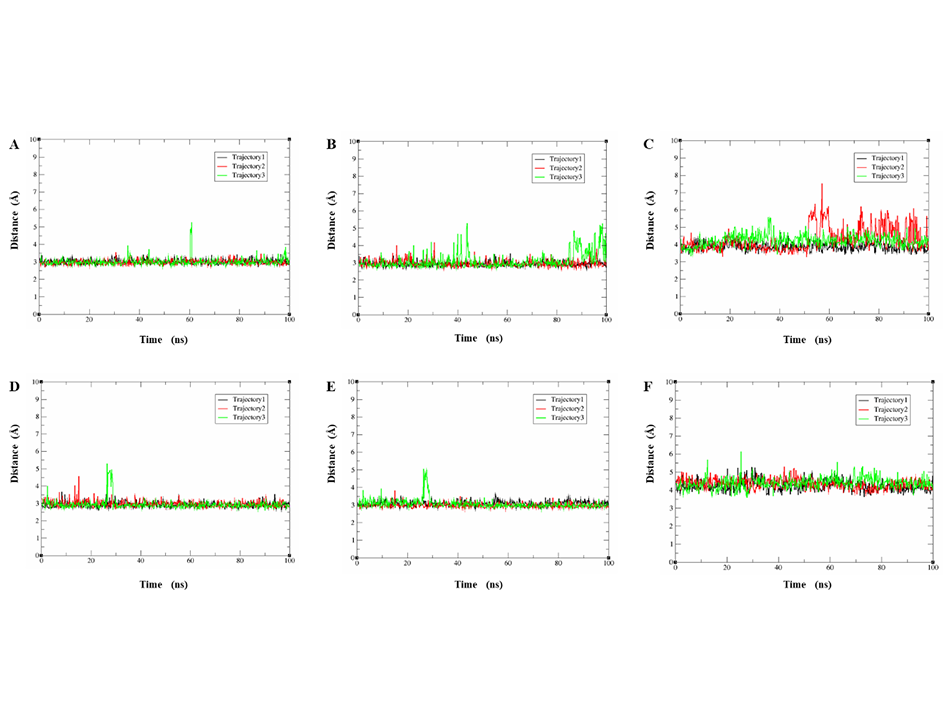

Supplement: S10 Fig — N-N distance between 1,2,4-triazol and the side chain amino group of N2546.55 in the C8-A1AR complex (A) and C9-A1AR complex (D). N-O distance between the 5-amino group and the side chain carbonyl of N2546.55 in the C8-A1AR complex (B) and C9-A1AR complex (E). Distance between the centroids of 1,2,4-triazol and the side chain phenyl of F171ECL2 in the C8-A1AR complex (C) and C9-A1AR complex (F). (TIF) [file pcbi.1008821.s010.tif]

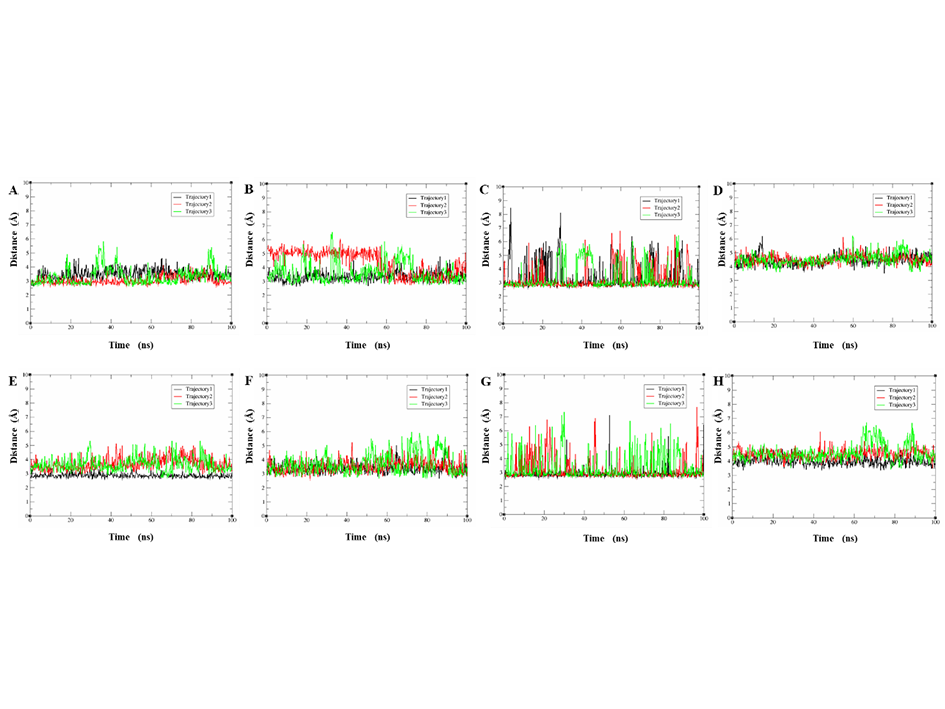

Supplement: S11 Fig — O-N distance between methanone and the side chain amino group of N2536.55 in the C8-A2AAR complex (A) and C9-A2AAR complex (E). N-O distance between the 5-amino group and the side chain carbonyl of N2536.55 in the C8-A2AAR complex (B) and C9-A2AAR complex (F). N-O distance between the 5-amino group and side chain of E169ECL2 in the C8-A2AAR complex (C) and C9-A2AAR complex (G). Distance between the centroids of 1,2,4-triazol and the side chain phenyl of F168ECL2 in the C8-A2AAR complex (D) and C9-A2AAR complex (H). (TIF) [file pcbi.1008821.s011.tif]

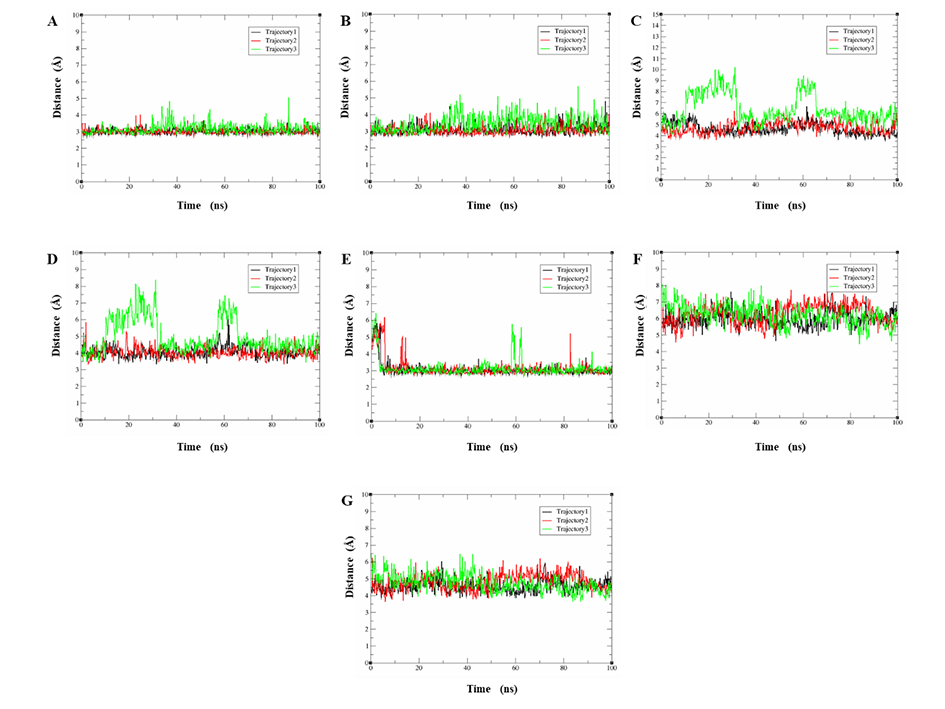

Supplement: S12 Fig — N-N distance between pyrazolo[1,5-a]pyrimidin and the side chain amino group of N2546.55 (or N2536.55) in the C10-A1AR complex (A) and C10-A2AAR complex (E). N-O distance between the N-propyl group and the side chain carbonyl of N2546.55 in the C10-A1AR complex (B). Distance between the centroids of pyrazole and the side chain phenyl of F171ECL2 (or F168ECL2) in the C10-A1AR complex (C) and C10-A2AAR complex (F). Distance between the centroids of pyrimidine and the side chain phenyl of F171ECL2 (or F168ECL2) in the C10-A1AR complex (D) and C10-A2AAR complex (G). (TIF) [file pcbi.1008821.s012.tif]

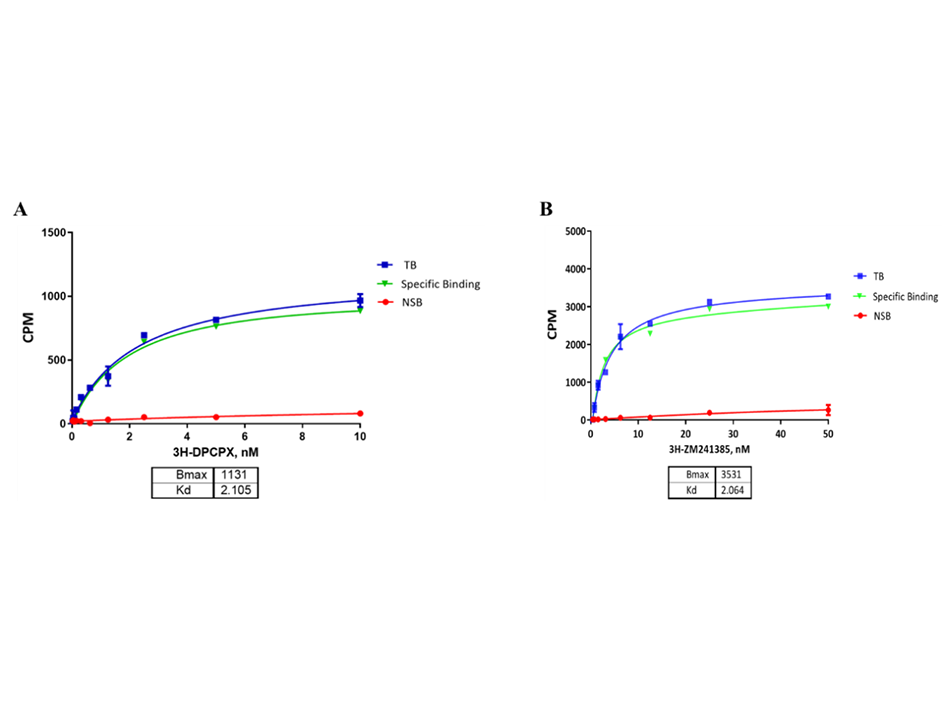

Supplement: S13 Fig — (A) Kd determination curves of [3H]DPCPX against A1AR in the filtration binding assay. Nonspecific signal: Different ligand concentrations of 10 μM DPCPX; (B) Kd determination curves of [3H]ZM241385 against A2AAR in the saturation binding assay. Nonspecific signal: Different ligand concentrations with 10 μM ZM241385. CPM = counts per minute, TB = total binding, NSB = nonspecific binding. (TIF) [file pcbi.1008821.s013.tif]

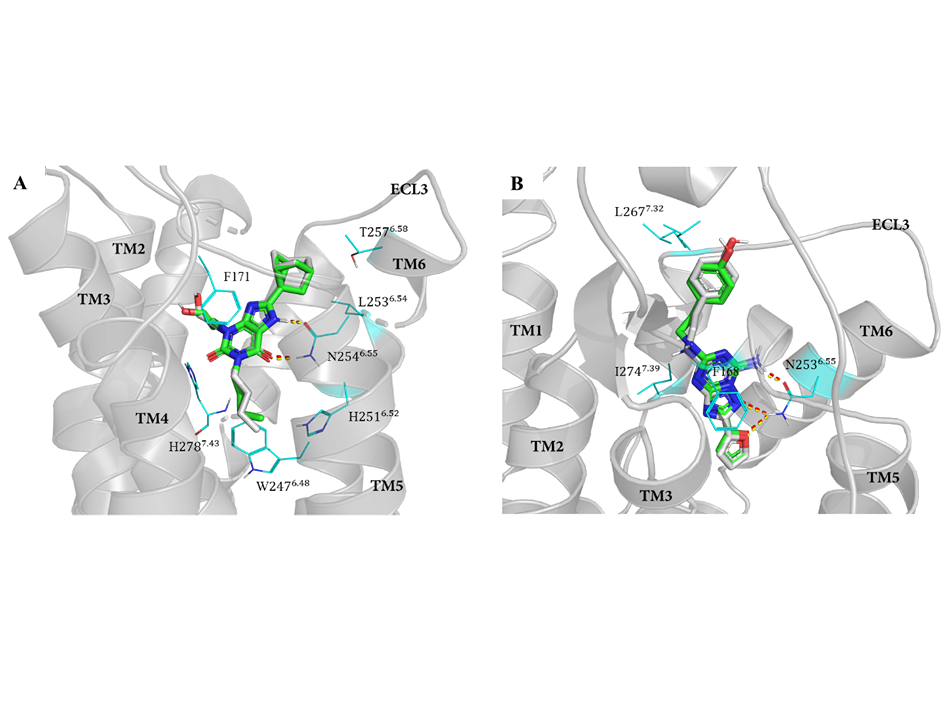

Supplement: S14 Fig — (A) Superposition of PSB36 in the orthosteric binding area of A1AR at the experimental structure (shown as sticks in white color) and the resulting docking pose (in green). (B) Superposition of ZM241385 in the orthosteric binding area of A2AAR at the experimental structure (shown as sticks in white color) and the resulting docking pose (in green). The protein is shown as a gray cartoon. The hydrogen bonds are represented by dashed lines. The side chains of F171, W2476.48, H2516.52, L2536.54, N2546.55, T2576.58 and H2787.43 (F168, N2536.55, L2677.32 and I2747.39 in A2AAR) are represented as lines. (TIF) [file pcbi.1008821.s014.tif]
