# Supplementary material for: Discovery of novel dual adenosine A1/A2A receptor antagonists using deep learning, pharmacophore modeling and molecular docking
Source: PLoS Comput Biol. 2021 Mar 19;17(3):e1008821. doi: 10.1371/journal.pcbi.1008821 (PMC7978378; doi:10.1371/journal.pcbi.1008821)
Supplement: S1 Table — (PDF) [file pcbi.1008821.s015.pdf]

S1 Table. Training set for the dual A_1/A_{2A} AR antagonist pharmacophore models.

Compound number	Compound ID	Chemical structures	K_i (nM)	
			A_1	A_{2A}
1	CHEMBL1095822	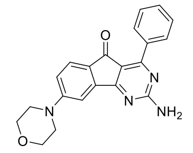	32.4	2.9
2	CHEMBL3121720	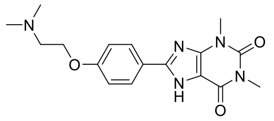	1.82	0.4
3	CHEMBL273094	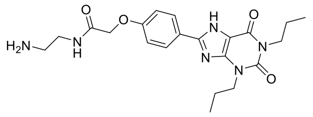	10.50	19.34
4	CHEMBL401321	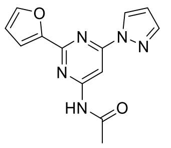	9.45	0.6
5	CHEMBL144979	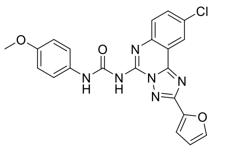	7.6	9.4
6	CHEMBL196258	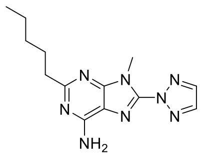	26.2	3.3
7	CHEMBL122622	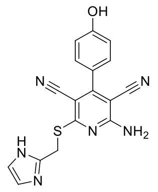	2.6	28
8	CHEMBL485765	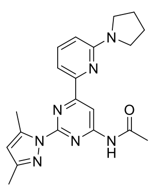	7.2	0.26
9	CHEMBL2419148	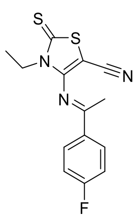	0.00059	10.98

10	CHEMBL596014	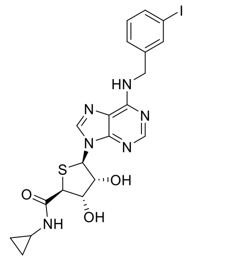	9.27	15.2
11	CHEMBL262363	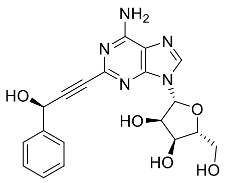	0.67	1.8
12	CHEMBL461857	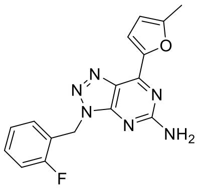	19.0	2.1
13	CHEMBL284969	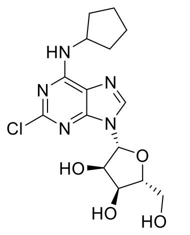	6.94	17
14	CHEMBL464859	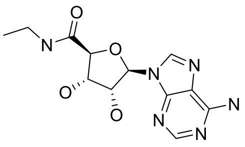	11.22	17.20
