# Supplementary material for: Discovery of novel dual adenosine A1/A2A receptor antagonists using deep learning, pharmacophore modeling and molecular docking
Source: PLoS Comput Biol. 2021 Mar 19;17(3):e1008821. doi: 10.1371/journal.pcbi.1008821 (PMC7978378; doi:10.1371/journal.pcbi.1008821)
Supplement: S2 Table — (PDF) [file pcbi.1008821.s016.pdf]

S2 Table. Performance of various VS methods by screening the validation set with 433 dual A_1/A_{2A} AR antagonists and 11,605 decoys.

Method	Predicted dual antagonists	Hits	Hit rate (%)	Time (h)
Deep learning[a]	1034	158	15.28	0.37
Pharmacophore	7970	299	3.75	3.68
Docking	66	23	34.85	57.58
Deep learning-pharmacophore	1034/607	158/127	20.92	0.65
Deep learning-docking	1034/70	158/26	37.14	8.7
Deep learning-pharmacophore-docking	1034/607/53	158/127/21	39.62	4.87

[a] combination of DNN and CNN.
