# Supplementary material for: Discovery of novel dual adenosine A1/A2A receptor antagonists using deep learning, pharmacophore modeling and molecular docking
Source: PLoS Comput Biol. 2021 Mar 19;17(3):e1008821. doi: 10.1371/journal.pcbi.1008821 (PMC7978378; doi:10.1371/journal.pcbi.1008821)
Supplement: S3 Table — (PDF) [file pcbi.1008821.s017.pdf]

S3 Table. Tc values of compounds C8 and C9 for Morgan, ECFP4 and MACCS.

Compound	Morgan	ECFP4	MACCS
C8	0.39	0.38	0.88
C9	0.36	0.38	0.84
