# Supplementary material for: Discovery of novel dual adenosine A1/A2A receptor antagonists using deep learning, pharmacophore modeling and molecular docking
Source: PLoS Comput Biol. 2021 Mar 19;17(3):e1008821. doi: 10.1371/journal.pcbi.1008821 (PMC7978378; doi:10.1371/journal.pcbi.1008821)
Supplement: S4 Table — (PDF) [file pcbi.1008821.s018.pdf]

S4 Table. RMSD between the redocked and crystallized conformations of PSB36 and ZM241385 by Glide XP.

Ligand	RMSD (Å)
PSB36	1.39
ZM241385	1.70
